# The prevalence of latent tuberculosis infection in patients with chronic kidney disease: A systematic review and meta-analysis

**DOI:** 10.1016/j.heliyon.2023.e17181

**Published:** 2023-06-10

**Authors:** Ayinalem Alemu, Zebenay Workneh Bitew, Getu Diriba, Getachew Seid, Shewki Moga, Saro Abdella, Emebet Gashu, Kirubel Eshetu, Getachew Tollera, Mesay Hailu Dangisso, Balako Gumi

**Affiliations:** a Ethiopian Public Health Institute, Addis Ababa, Ethiopia; b Aklilu Lemma Institute of Pathobiology, Addis Ababa University, Addis Ababa, Ethiopia; c St. Paul's Hospital Millennium Medical College, Addis Ababa, Ethiopia; d Addis Ababa Health Bureau, Addis Ababa, Ethiopia; e USAID Eliminate TB Project, Management Sciences for Health, Addis Ababa, Ethiopia

**Keywords:** Latent tuberculosis, Chronic kidney disease, Dialysis, Hemodialysis, Renal transplant, Peritoneal dialysis

## Abstract

**Objective:**

To estimate the prevalence of latent tuberculosis infection (LTBI) in chronic kidney disease (CKD) patients**.**

**Methods:**

This study was conducted following the PRISMA guidelines. We identified, 3694 studies from the whole search, and 59 studies were included. Each study's quality was assessed using JBI checklist. We employed STATA version 17 for statistical analysis. We assessed heterogeneity using I^2^ heterogeneity test. Publication bias was assessed using funnel plot and Egger's test. We estimated the pooled LTBI prevalence in CKD patients along with 95%CI.

**Results:**

The pooled prevalence of LTBI among CKD patients using data collected from 53 studies having 12,772 patients was 30.2% (95%CI; 25.5, 34.8). The pooled prevalence among pre-dialysis, hemodialysis, peritoneal dialysis, and renal transplanted patients was 17.8% (95%CI; 3.3, 32.4), 34.8% (95%CI; 29.1, 40.5), 25% (95%CI; 11, 38), and 16% (95%CI; 7, 25), respectively. The pooled prevalence of LTBI stratified by the laboratory screening methods was 25.3% (95%CI: 20.3–30.3) using TST, 28.0% (95%CI; 23.9–32.0) using QFT, and 32.6%, (95%CI: 23.7–41.5) using T-SPOT.

**Conclusion:**

There is high prevalence of LTBI among CKD patients mainly in patients on dialysis. Thus, early diagnosis and treatment of LTBI in CKD patients should be performed to prevent active TB in CKD patients.

**PROSPERO registration number:** CRD42022372441.

## 1 Introduction

Tuberculosis (TB) continues to be a major public health issue across the globe. It is the second leading cause of mortality among infectious diseases next to COVID-19 [1]. Countries are committed to control and prevent TB by developing and adapting different strategies and measurable targets. The World Health Organization (WHO) developed the END-TB strategy that aims to reduce the incidence of TB to less than 10 per 100,000 populations by 2035 [2]. However, achieving this ambitious objective may be challenging unless a specific intervention approach that addresses the burden in a high-risk population is created and implemented. For example, specific groups of people, such as those with chronic kidney disease (CKD), are at a higher risk of contracting TB than the overall population, necessitating a focused intervention [3]. According to our recent global systematic review and meta-analysis, the incidence of TB in CKD patients was 3718/100,000 population [4]. A pooled estimate revealed that patients on dialysis had 3.6 times the risk to develop TB compared to the general population [5]. Currently, the incidence of CKD is rising in developing countries where TB is also endemic which may halt the TB prevention and control efforts to achieve the END-TB strategy [6].

Early detection and treatment of latent tuberculosis infection (LTBI) among groups of people with weakened immune systems, such as CKD patients, is critical for preventing the development of active TB. In addition, dialysis patients frequently travel to health facilities for medical care, which may increase the risk of infection with *Mycobacterium tuberculosis* [7]. When compared to healthy adults, these patients have a 10–25 fold increased chance of risk of reactivating LTBI [7]. The WHO recommends that persons undergoing dialysis or preparing for an organ transplant be tested and treated for LTBI [8]. There have been studies undertaken in different countries and settings to determine the prevalence of LTBI in CKD patients [7,[9], [10], [11], [12], [13], [14], [15], [16], [17], [18], [19], [20], [21], [22], [23]]. The prevalence of LTBI in CKD patients has been found to range from 6% [24,25] to 82% [23]. The systematic reviews were primarily concerned with comparing the performance of diagnostic tools for detecting of LTBI in dialysis patients [[26], [27], [28]]. However, there is limited data that reported the global, and regional prevalence of LTBI among CKD patients in general and across different categories. A global data that comprehensively assessed the burden of LTBI in CKD patients can be an essential input for policy development and guidance to boost the effort for TB prevention and control, as well to improve the quality of life for this population group. Thus, this study aimed to estimate the global pooled prevalence of LTBI among patients with CKD.

## 2 Methods

### 2.1 Protocol registration

The protocol for this systematic review and meta-analysis study is registered on the International Prospective Register of Systematic Reviews (PROSPERO) with a registration number CRD42022372441.

### 2.2 Article search strategy and selection procedure

**Fig. 1:**
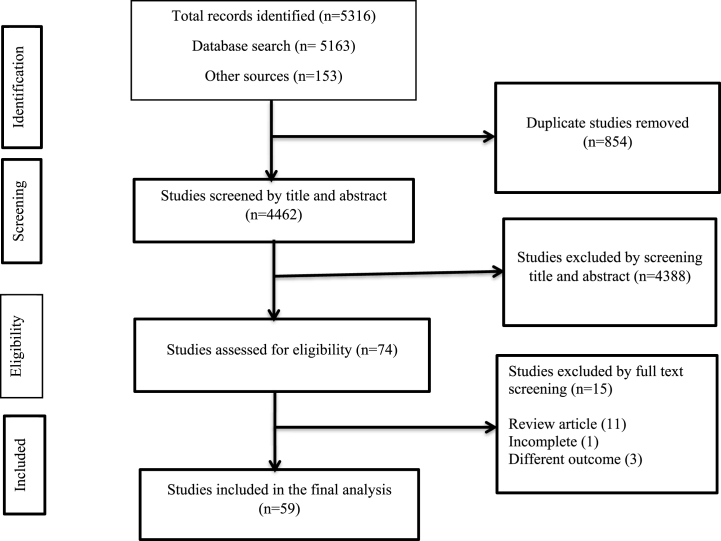
Flowchart describing the selection of studies for the systematic review and meta-analysis of latent tuberculosis prevalence among patients with chronic kidney disease.

This systematic review and meta-analysis study followed the Preferred Reporting Items for Systematic Reviews and Meta-Analyses (PRISMA) guidelines [29]. Two independent investigators (AA, ZWB) conducted article searching, and the third investigator (GD) resolved the inconsistencies. Both electronic databases and grey literature sources were searched for previously published studies that reported LTBI among patients with any types of CKDs. We searched articles published in English language until November 21, 2022. PubMed, Global Index Medicus, Informit, Joanna Briggs Institute EBP Database (including OVID), and Global Health were among the electronic databases used. Whereas the grey literature sources were Google, and Google Scholar. The searching was carried out using the key terms in conjunction with the Boolean operators AND and OR. The keywords used in the current study includes; latent tuberculosis, chronic kidney disease patient, renal failure, dialysis, hemodialysis, peritoneal dialysis, renal-transplant, pre-renal transplant, and pre-dialysis. All of the articles identified during the entire search were exported to Endnote X8 citation manager. We have followed a stepwise approach to select the studies included in the final data-analysis. In the primary step, duplicates were removed and then the articles were screened by title and abstract. All the articles that passed the above stage were eligible for full-text screening and those that passed the full-text assessment were included in the final data analysis (Fig. 1) (Appendix).

### 2.3 PICOS criteria

Participants: Patients with chronic kidney disease.

Intervention: Not applicable.

Comparator: Not applicable.

Outcome: Latent TB infection.

Study design: Observational studies.

Study setting: Any setting in any country across the globe.

### 2.4 Inclusion and exclusion criteria

Studies that assessed prevalence of LTBI among different categories of CKD patients (pre-dialysis, hemodialysis, peritoneal dialysis, or renal transplanted) were included in the study. Review studies, incomplete studies and articles with different outcomes were excluded.

### 2.5 Data extraction

We extracted data from all studies included in the current systematic review and meta-analysis using the 2016 Microsoft Excel Spreadsheet. Two investigators (GS, EG) extracted data independently, and the inconsistencies were resolved through discussion and consensus was reached with the guidance of the third author (AA). The extracted data included; first author name, publication year, country, data collection period, study design, age group, type of CKD patients included, laboratory screening method, sample size, and number of patients who had LTBI. In addition to the above variables, the studies were categorized based on continent, WHO regional classification, and country TB burden category **(**Table 1**).**

**Table 1:** Characteristics of individual studies on the prevalence of latent tuberculosis among patients with chronic kidney disease, included in the current systematic review and meta-analysis.

Author year	Country	Continent	TB burden category	Study period	Study design	Study group	Study setting	Age group	Diagnostic method
Wu et al., 2021	Taiwan	Asia	Not HBC	September 5, 2018 to September 5, 2019	Prospective study	HD	Kaohsiung Chang Gung Memorial Hospital	>18 years	QFT
Shu et al., 2015	Taiwan	Asia	Not HBC	January 2012 to June 2013	Cross-sectional	Patients undergoing dialysis, and those with severe CKD	National Taiwan University Hospital, a tertiary referral center, and its branches, regional teaching hospitals, and a local hemodialysis clinic.	≥20 years	QFT
Shu et al., 2012	Taiwan	Asia	Not HBC	March 2011 to February 2012	Cross-sectional	Dialysis	National Taiwan University Hospital, a tertiary referral center in northern Taiwan, and its branch in southern Taiwan.	≥20 years	QFT
Fonseca et al., 2013	Brazil	South America	HBC	December of 2008 to December of 2009	Cross-sectional	HD	Mineiro Institute of Nephrology	>18 years	TST
Gunluoglu et al., 2015	Turkey	Asia	Not HBC	September to November 2011	Cross-sectional	HD	Yedikule Chest Diseases and Thoracic Surgery Training and Research Hospital.	Mean age 62.2 years	TST and QFT
Passalent et al., 2007	Canada	North America	Not HBC	January 15 to April 15, 2005	Cross-sectional	HD	Toronto General Hospital site of the University Health Network	All	TST and T-SPOT
Ferreira et al., 2021	Brazil	South America	HBC	July to December 2018	Cross-sectional	HD	Clinical Hospital (HCFMRP-USP), of the Ribeirão Preto Nephrology Service (SENERP)	≥18 years	TST
Setyawati et al., 2021	Indonesia	Asia	HBC	May 2018	Cross-sectional	HD	Dr. Moewardi Surakarta Hospital	>18 years	TST and T-SPOT
Lee et al., 2010	Taiwan	Asia	Not HBC	October 2008	Cross-sectional	HD	Kaohsiung Veterans General Hospital	16.8–93.5 years	TST and QFT
Ahmadinejad et al., 2012	Iran	Asia	Not HBC	October 2009 to November 2010	Cross-sectional	Pre-transplantation	Tehran University of Medical Sciences.	16–65 years	TST and QFT
Sester et al., 2004	Germany	Europe	Not HBC	December 2001 to December 2002	Cross-sectional	HD	University of the Saarland	Mean age was 61.2 ± 15.2 years	TST
Al Jahdali et al., 2013	Saudi Arabia	Asia	Not HBC	August to December 2010	Cross-sectional	HD	KAMC-R	Mean age was 62.27 ± 11.79 years	TST and QFT
Kim et al., 2011	South Korea	Asia	Not HBC	June 2008 to December 2009	Cross-sectional	Transplant	University of Ulsan College of Medicine	>16 years	TST
Al Wakeel et al., 2015	Saudi Arabia	Asia	Not HBC	January 5, 2011 to March 31, 2013	Prospective study	Dialysis	King Khalid University Hospital, Security Forces Hospital and Lehbi Medical Center	≥18 years	TST and QFT
Lee et al., 2015	South Korea	Asia	Not HBC	–	Prospective study	Dialysis	Inje University Busan Paik hospital.	23–74 years	QFT
Ates et al., 2010	Turkey	Asia	Not HBC	15 February to July 15, 2008	Cross-sectional	HD	13 hemodialysis centers in five different cities	>15 years	TST
Chung et al., 2009	South Korea	Asia	Not HBC	1 March to April 30, 2008	Cross-sectional	HD	Gil Medical Centre, Gachon University of Medicine and Science	17–88 years	TST, QFT and T-SPOT
Shu et al., 2019	Taiwan	Asia	Not HBC	2014 to 2018	Cross-sectional	On pre transplantation and after transplantation	National Taiwan University Hospital,	≥20 years	QFT
Agarwal et al., 2015	India	Asia	HBC	May 2007 to August 2010	Cross-sectional	HD	All India Institute of Medical Sciences	18–88 years	TST and QFT
Sultan et al., 2016	Iraq	Asia	Not HBC	1st of April to December 15, 2014	Cross-sectional	HD	Baghdad teaching hospital	Mean age was 54.34 ± 15.25 years	TST
Hassen et al., 2013	Saudi Arabia	Asia	Not HBC	January 1 to May 30, 2009	Cross-sectional	HD	King Fahad specialist hospital in Dammam	≥18 years	TST and T-SPOT
Chagas et al., 2014	Brazil	South America	HBC	July 2011 to December 2013	Cross-sectional	HD	six existing dialysis services in Campo Grande, MS, Brazil	>18 years	TST
Romanowski., 2020	British Columbia, Canada	North America	Not HBC	January 1, 2012 to May 31, 2017	Retrospective cohort	Dialysis	British Columbia	≥18 years	IGRA (Not specified)
Hussein et al., 2017	Egypt	Africa	Not HBC	February to April 2016	Prospective study	HD	Sohag University Hospital	21–65 years	TST and QFT
Shankar et al., 2005	India	Asia	HBC	–	Prospective study	ESRD	Postgraduate Institute of Medical Education and Research Hospital, Chandigarh	18–60 years	TST
Triverio et al., 2009	Switzerland	Europe	Not HBC	–	Cross-sectional	HD	Geneva University Hospital	Mean age was 65 ± 15 years	TST, QFT and T-SPOT
Soysal et al., 2012	Turkey	Asia	Not HBC	May 2006 to May 2007	Prospective study	HD	Marmara University School of Medicine	19–84 years	TST and T-SPOT
Habesoglu et al., 2007	Turkey	Asia	Not HBC	–	Prospective study	HD	Adana Teaching and Training Hospital of Baskent University	Mean age was 50.0 ± 15.9 years	TST
Hoffmann et al., 2010	Switzerland	Europe	Not HBC	–	Prospective study	HD	Kantonsspital, St.Gallen	≥18 years	TST and QFT
Agarwal et al., 2010	India	Asia	HBC	May 2000 to May 2006	Prospective study	Transplant	All India Institute of Medical Sciences	14–60 years	TST
Lee et al., 2009	Taiwan	Asia	Not HBC	September 2005	Prospective study	HD	Kaohsiung Veterans General Hospital	34.4–77.7 years	TST, QFT and T-SPOT
Lin et al., 2020	Taiwan	Asia	Not HBC	March 1, 2017, to May 31, 2017	Cross-sectional	HD	Kaohsiung Medical University Hospital	≥20 years	QFT
Baek et al., 2019	South Korea	Asia	Not HBC	March 2017 to August 2019	Cross-sectional	Dialysis	Mediplex Sejong Hospital	All (mean age was 61.6 ± 12.6 years)	QFT
Bandiara et al., 2021	Indonesia	Asia	HBC	March to May 2020	Cross-sectional	HD	Dr. Hasan Sadikin Hospital	≥18 years	IGRA (Not specified)
Ogawa et al., 2021	Japan	Asia	Not HBC	–	Cross-sectional	HD	3 hospitals	62–79 years	QFT
SAYARLIOĞLU et al., 2011	Turkey	Asia	Not HBC	–	Cross-sectional	HD	Kahramanmaras State Hospital,	Mean age was 54.6 ± 14.9 years	TST and QFT
Carrazco-Ibarra et al., 2017	Mexico	North America	Not HBC	2011–2016	Retrospective cohort	Pre-transplantation	Hospital based study	–	TST
Seyhan et al., 2009	Turkey	Asia	Not HBC	November 2008 to December 2008	Cross-sectional	HD	Yedikule Chest Diseases and Thoracic Surgery Education and Research Hospital	Mean age was 56.2 ± 15.3 years	TST and QFT
Wauters et al., 2004	Belgium	Europe	Not HBC	September–October 2001	Cross-sectional	HD	University Hospital Gasthuisberg KU Leuven, Leuven and Virga Jesse Hospital, Hasselt	21–92 years	TST
Fang et al., 2002	Taiwan	Asia	Not HBC	June to July 1999	Cross-sectional	Dialysis	Kaoh siung Veterans General Hospital.	Mean age was 54.7 ± 17.3 years	TST
Winthrop et al., 2008	USA	North America	Not HBC	Oct-03	Cross-sectional	HD	Multisite study conducted by USA CDC	18–90 years	TST, QFT, and ELISPOT
Woeltje et al., 1998	USA	North America	Not HBC	June 1996 to August 1996	Cross-sectional	HD	Washington University School of Medicine, St Louis	19–91 years	TST
Smirnoff et al., 1998	USA	North America	Not HBC	1995	Cross-sectional	HD	The Mount Sinai Medical Center,	19–89 years	TST
Connel et al., 2010	United Kingdom	Europe	Not HBC	2008	Cross-sectional	CKD with different categories	Hammersmith Hospital	28–88 years	TST, QFT and T-SPOT
Cengiz et al., 2005	Turkey	Asia	Not HBC	–	Cross-sectional	HD	Ondokuz Mayıs University School of Medicine	Mean age was 49.9 ± 14.4 years	TST
Akcay et al., 2003	Turkey	Asia	Not HBC	January 1 to December 31, 1999	Cross-sectional	HD	Hacettepe University School of Medicine, Hemodialysis Unit	20–72 years	TST
Dogan et al., 2005	Turkey	Asia	Not HBC	June to December 2003	Prospective study	HD	Van (Yuzuncu Yil University Hospital, Yuksek Ihtisas Hospital) and the Mus State Hospital).	13–82 years	TST
Foster et al., 2016	Canada	North America	Not HBC	February 2008 to December 2008	Retrospective cohort	Dialysis	4 major hospital dialysis units in Winnipeg, Manitoba.	Mean age was 54.3 ± 14.7 years	TST
Khosroshahi et al., 2012	Iran	Asia	Not HBC	–	Cross-sectional	HD	two university hospitals in Tabriz	Mean age was 44.6 ± 15 years	TST
Altunoren et al., 2012	Turkey	Asia	Not HBC	–	Cross-sectional	Dialysis	Kahramanmara¸s Sutcu Imam University	Mean age was 51.9 ± 15.5 years	TST
Edathodu et al., 2016	Saudi Arabia	Asia	Not HBC	August 2008 to May 2013	Prospective study	ESRD	King Faisal Specialist Hospital and Research Centre	≥14 years	TST and QFT
Maciel et al., 2018	Brazil	South America	HBC	January 2011 to July 2013	Cross-sectional	Transplant	Federal University of Minas Gerais Hospital das Clínicas	≥18 years	TST
Igari et al., 2019	Japan	Asia	Not HBC	April 2017 to March 2018	Cross-sectional	Transplant	National Hospital Organization Chiba-East Hospital	20–79	QFT and T-SPOT
Meinerz et al., 2021	Brazil	South America	HBC	April 4th, 2014 to Oct 31st, 2018, follow-up until Oct 31st, 2019	Prospective study	Transplant	Hospital based study	18–80 years	TST and QFT
Grant et al., 2012	Canada	South America	Not HBC	–	Cross-sectional	HD	Vancouver General Hospital	≥18 years	TST, QFT and T-SPOT
Chung et al., 2010	South Korea	Asia	Not HBC	February 1st to March 31st, 2009	Cross-sectional	HD	Gil Medical Center	18–81 years	TST, QFT and T-SPOT
Mohtashami et al., 2022	Iran	Asia	Not HBC	2018	Cross-sectional	HD	Khorramabad teaching hospitals.	>15 years	TST
Wang et al., 2020	Taiwan	Asia	Not HBC	2016–2019	Cross-sectional	HD	two tertiary-care medical centers	Mean age was 56.7 ± 11.2 years	QFT
Harris et al., 2016	Canada	North America	Not HBC	2007 to 2014	Cross-sectional	ESRD	British Columbia Centre for Disease Control	All	TST and IGRA (Not specified)

CKD; Chronic Kidney Disease, HD; Hemodialysis, HBC; High TB Burden Country, TST; Tuberculin Skin Test, QFT; QuantiFERON®-TB Gold, ELISPOT; enzyme-linked immunospot, “-“; Not described.

### 2.6 Outcome

The primary outcome of this study was detection of LTBI among CKD patients with any category including pre-dialysis, hemodialysis, peritoneal dialysis, and renal transplant. The included studies used different laboratory screening methods alone or in combination for screening of LTBI in CDK patients; Tuberculin Skin Test (TST), QFT (QuantiFERON®-TB Gold), T-SPOT, and ELISPOT (enzyme-linked immunospot). The TST result was considered positive when the cut-off induration was ≥10 mm. From studies that employed a two-step TST, only the baseline results were taken to avoid a boosting phenomenon.

### 2.7 Quality assessment

Two independent investigators (AA, GS) assessed the quality and validity of individual studies included in this study using the Joanna Brigg's Institute critical appraisal tool [30]. The inconsistencies that arose between the two authors were resolved by the third investigator (ZWB). The JBI tool for prevalence study was used to assess the study's quality. Each question on the checklist was scored equally, and their total was calculated out of 100%. We classified the quality score as low, medium, and high quality when the score was <60%, 60–80%, and >80%, respectively.

### 2.8 Data synthesis and analysis

The data that were summarized in the 2016 Microsoft Excel Spreadsheet were exported to STATA version 17 for statistical analysis. The pooled prevalence of LTBI among CKD patients was estimated along with 95%CI. Sub-group analysis was performed based on CKD categories, LTBI laboratory screening methods, WHO regional classification, continent, country's income level, publication year, and TB burden category. We have presented the pooled estimates using forest plot. The presence of heterogeneity among studies was assessed using I^2^ heterogeneity test where I^2^ >50% was considered as the presence of substantial heterogeneity [31,32]. We have used the random-effect model considering the presence of substantial heterogeneity. We assessed the presence of publication bias through the visual inspection of the funnel plot and the statistical significance of the Egger's regression test (P < 0.05) [33,34]. A trim-and fill analysis was done to adjust the publication bias [35].

## 3 Results

### 3.1 Study characteristics

From the whole search, 5316 studies were identified and 854 duplicates were removed. Title and abstract screening was conducted for the 4462 studies and 4388 were excluded. Then, the remaining 74 studies were assessed for full text and 59 studies were included in the final analysis. While the remaining 15 studies were excluded due to different reasons (review articles, incomplete studies, articles with different outcomes) (Fig. 1) (Appendix).

The studies were conducted in 18 countries from four continents. The highest number of studies were from Asia (39 studies) followed by North America (8 studies), South America (6 studies), and Europe (5 studies). The least number of study was from Africa with only one study conducted in Egypt. Based on the WHO regional classifications, the highest number of studies were from the West Pacific Region (WPR) with 16 studies followed by the European Region (EUR) (15 studies), the Region of Americans (AMR) (14 studies), Eastern Mediterranean Region (EMR) (9 studies), and South East Asian Region (SEAR) (5 studies). No study was reported from the WHO African Region (AFR). Specifically, the most frequent studies were from Turkeye (10 studies) followed by Taiwan (9 studies), South Korea (5 studies), Canada (5 studies), and Brazil (5 studies). The other studies were reported from Saudi Arabia, United States, India, Iran, Indonesia, Japan, Switzerland, Belgium, Egypt, Germany, Iraq, Mexico, and United Kingdom. Based on the World Bank income classification, 6, 20, and 27 studies were reported from lower middle income, upper middle income and high income countries, respectively.

Based on publication year, 22, 17, and 20 studies were published from 1998 to 2010, from 2011 to 2015 and from 2016 to 2022. Based on the 2021 global TB report, 10 studies were reported from high TB burden countries while the remaining 49 studies were reported from countries that were not included in the high TB burden country list. The majority of the studies (43 studies) were conducted using a cross-sectional study design **(**Table 1**)**.

Different laboratory diagnostic methods such as TST and IGRA (QFT, T-SPOT and ELISPOT) were used to detect LTBI in CKD patients. The IGRA method was used in 36 studies where QFT, T-SPOT, and ELISPOT were used in 29, 11 and 1 studies, respectively. However, the type of IGRA methods were not specified in three studies. Tuberculin skin test was used in 47 studies. These diagnostic methods were used alone or in different combinations **(**Table 1**)**.

### 3.2 Pooled prevalence of latent tuberculosis among patients with chronic kidney disease

**Fig. 2:**
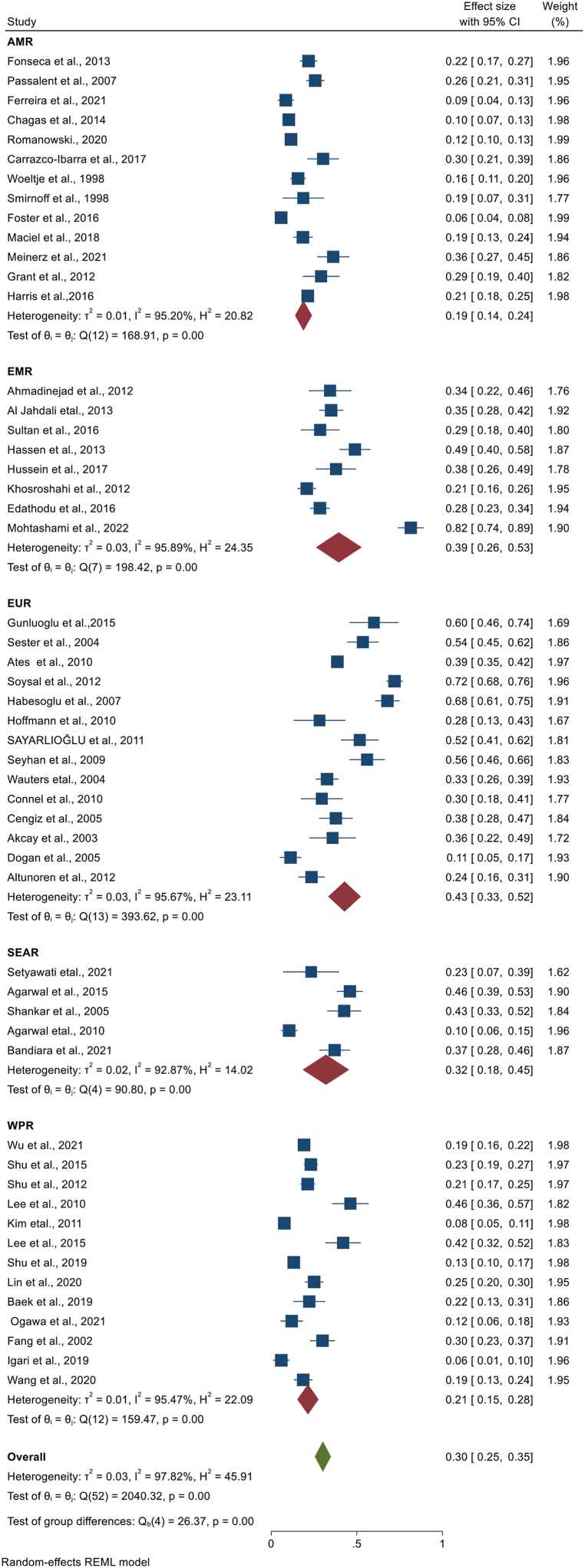
Forest plot for the pooled prevalence of latent tuberculosis among patients with chronic kidney disease.

**Fig. 3:**
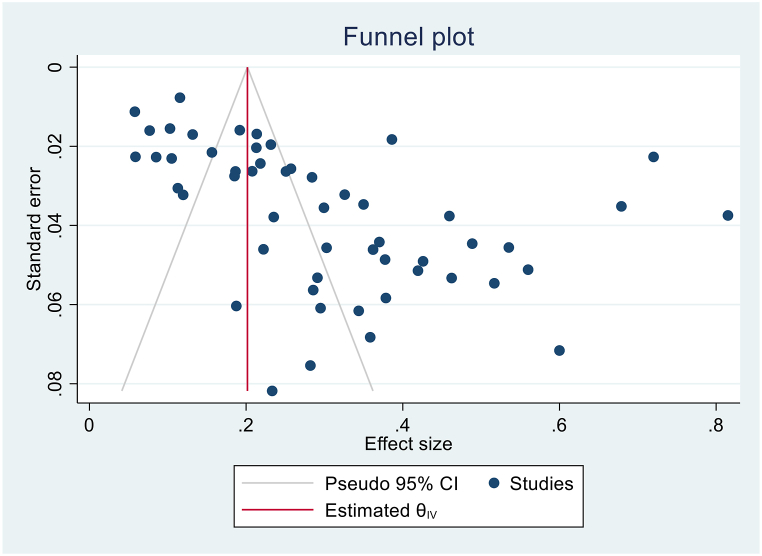
Funnel plot for the pooled the pooled prevalence of latent tuberculosis among patients with chronic kidney disease.

We have extracted data from 59 studies, but the pooled prevalence of LTBI among CKD patients was determined by using 53 studies. In the remaining six studies, the studies used two or more laboratory screening methods and data was available for the specific method, but we were unable to get the overall LTBI prevalence in combination of the laboratory methods. The highest sample size was 1790 [13], while the lowest sample size was 30 [36]. The highest prevalence was 82% from Iran [23], and the lowest prevalence 6% from Canada [24] and from Japan [25]. When pooled together, 3219 CKD patients had LTBI from 12,772 patients. Based on the random effect model, the pooled prevalence of LTBI among CKD patients was estimated as 30.2% (95%CI; 25.5, 34.8, I^2^; 97.82%) (Fig. 2). There was high heterogeneity among studies, and publication bias was revealed by funnel plot (Fig. 3) and Egger's regression test (P = 0.0006). However, after the trim-and-fill analysis, there was no change in the pooled estimate (Fig. 2) (Table 2).

**Table 2:** The summary of the pooled prevalence of latent tuberculosis among chronic kidney disease patients per different categories.

Latent tuberculosis prevalence among patients with chronic kidney disease across different categories		Number of studies	Sample size	Number of LTBI cases	Pooled LTBI prevalence	
					Estimate, 95%CI	Heterogeneity
						I^2^
Over all latent tuberculosis prevalence		53	12,772	3219	30% (26, 35)	97.82%
Per continent	Europe	4	451	170	36% (25, 48)	82.78%
	Asia	35	7273	2253	33% (27, 40)	97.57%
	North America	7	3662	536	18% (12, 24)	95.78%
	South America	6	1005	163	20% (12, 28)	93.50%
	Africa	1	74	28	38% (26, 49)	–
Per WHO regional classification	Region of Americas	13	4974	766	19% (14, 24)	95.20%
	European Region	8	1193	434	43% (33, 52)	95.67%
	East Mediterranean Region	14	2440	1113	39% (26, 53)	95.89%
	South East Asian Region	5	643	206	32% (18, 45)	92.87%
	West Pacific Region	13	3522	700	21% (15, 28)	95.47%
	African Region	–	–	–	–	–
Per publication year	1998–2010	17	2994	1008	34% (26, 42)	95.41%
	2011–2015	16	3647	1099	34% (25, 43)	97.65%
	2016–2022	20	6131	1112	24% (17, 31)	98.31%
Per high TB burden category	Included	10	1876	413	25% (16, 34)	95.81%
	Not included	43	10,896	2806	31% (26, 37)	98.02%
Per World Bank Group income classification of countries	Lower Middle Income	6	717	234	33% (21, 44)	90.82%
	Upper Middle Income	20	3839	1375	37% (28, 47)	97.89%
	High Income	27	8216	1610	24% (20, 29)	96.75%
Per laboratory diagnostic method	TST	47	8208	1966	25% (20, 30)	97.71%
	QFT	29	4821	1285	28% (24, 32)	90.83%
	T-SPOT	13	1412	595	33% (24, 42)	97.53%
	ELISPOT	1	97	27	–	–
Per chronic kidney disease categories	Pre-dialysis	3	432	61	18% (3, 32)	90.61%
	Hemodialysis	39	7534	2396	35% (29, 41)	96.87%
	Peritoneal dialysis	5	253	60	25% (11, 38)	83.48%
	Post-renal transplantation	6	1100	159	16% (7, 25)	94.26%

TST; Tuberculin Skin Test, QFT; QuantiFERON®-TB Gold, ELISPOT; enzyme-linked immunospot, WHO; World Health Organization, “-“; Not available.

Per continent, the highest pooled prevalence of LTBI among CKD patients was found in Europe (estimate; 36%, 95%CI; 25, 48, I^2^; 82.78%) followed by Asia (estimate; 33%, 95%CI; 27, 40, I^2^; 97.57%), South America (estimate; 20%, 95%CI; 12, 28, I^2^; 93.50%), and North America (estimate; 18%, 95%CI; 12, 24, I^2^; 95.78%). Since there is only one study from Africa conducted in Egypt with a prevalence of 38% (95% CI; 26, 49), we were unable to estimate the pooled prevalence. Per the WHO regional classification, the highest pooled estimate was found in EUR (estimate; 43%, 95%CI; 33, 52, I^2^; 95.67%) followed by EMR (estimate; 39%, 95%CI; 26, 53, I^2^; 95.89%), SEAR (estimate; 32%, 95%CI; 18, 45, I^2^; 92.87%), WPR (estimate; 21%, 95%CI; 15, 28, I^2^; 95.47%), and AMR (estimate; 19%, 95%CI; 14, 24, I^2^; 95.20%) (Fig. 2). In addition, we have also performed a sub-group analysis based on publication year. The pooled prevalence of LTBI among CKD patients based on studies published from 1998 to 2010, from 2011 to 2015, and from 2016 to 2022, were 34% (95%CI; 26%, 42, I^2^; 95.41%), 34% (95%CI; 25%, 43, I^2^; 97.65%), and 24% (95%CI; 17%, 31, I^2^; 98.31%), respectively. Besides, we performed a sub-group analysis considering classification of countries with TB burden category. Thus, the pooled prevalence of LTBI among CKD patients residing in high TB burden countries was 25% (95%CI; 16%, 34, I^2^; 95.81%), while the pooled LTBI prevalence among CKD patients residing in the countries not included in the list of high TB burden countries was 31% (95%CI; 26%, 37, I^2^; 98.02%). Furthermore, we have conducted a sub-group analysis based on the World Bank Group classification of countries by their income level**.** Accordingly, the pooled prevalence of LTBI was 24% **(**95%CI; 20%, 29, I^2^; 96.75%), 33% (95%CI; 21%, 44, I^2^; 90.82%), and 37% (95%CI; 28%, 47, I^2^; 97.89%) in CKD patients living in high income, lower middle income and upper middle income countries, respectively **(**Table 2**) (Appendix).**

### 3.3 Pooled prevalence of latent tuberculosis per laboratory diagnostic method

**Fig. 4:**
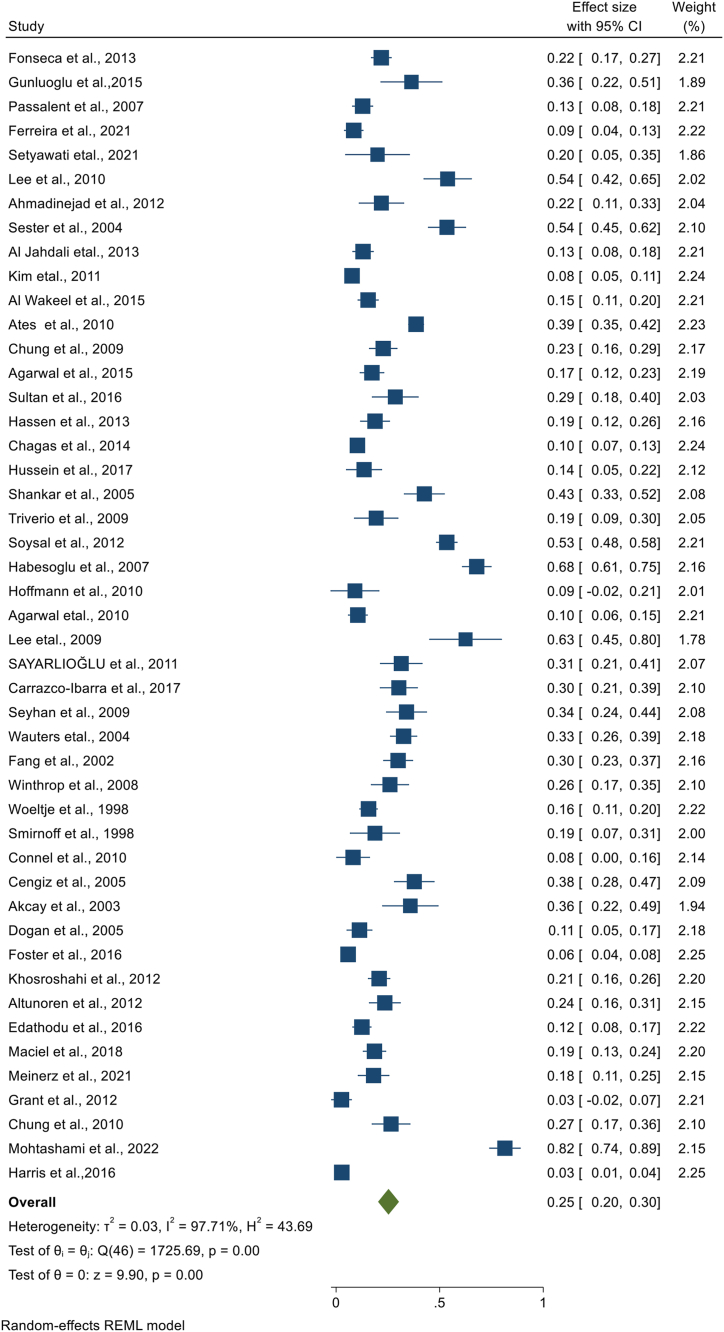
Forest plot for the pooled prevalence of latent tuberculosis among patients with chronic kidney disease diagnosed using TST.

**Fig. 5:**
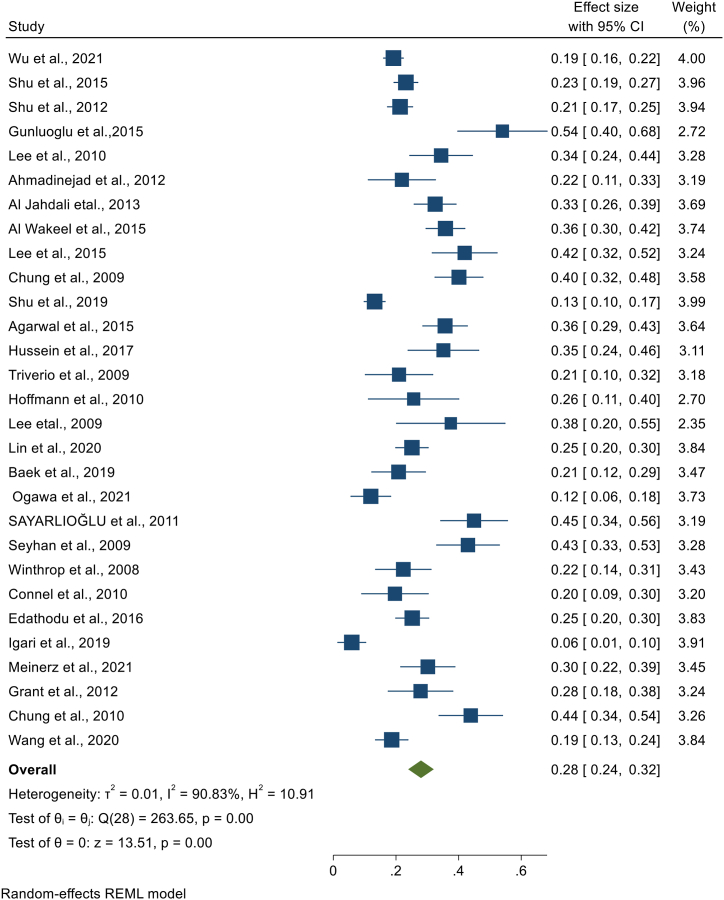
Forest plot for the pooled prevalence of latent tuberculosis among patients with chronic kidney disease diagnosed using QFT.

**Fig. 6:**
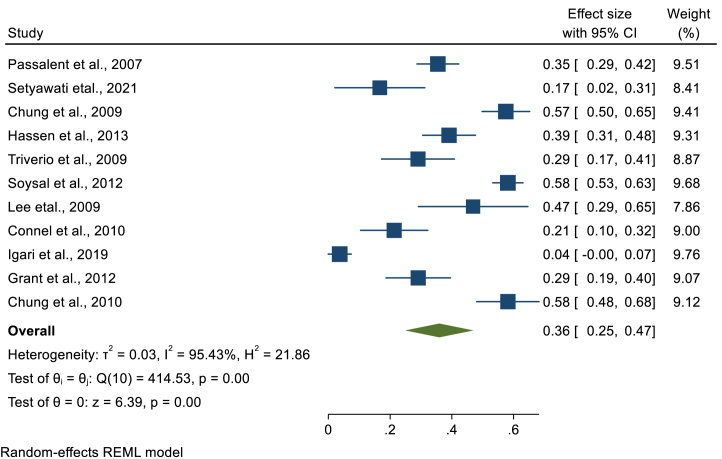
Forest plot for the pooled prevalence of latent tuberculosis among patients with chronic kidney disease diagnosed using TST.

We have performed a sub-group analysis, using the laboratory diagnostic method used to detect LTBI in CKD patients. Accordingly, TST, QFT, T-SPOT, ELISPOT, and unspecified IGRA were used. Tuberculin skin test was used by 47 studies, where the highest prevalence was 82% [23] and the lowest prevalence was 3% [37,38]. A total of 8208 CKD patients were screened by TST and 1966 were found to have LTBI. Based on the random effect model, the pooled prevalence of LTBI among CKD patients screened by TST was 25.3% (95%CI; 20.3%, 30.3%, I^2^; 97.71%) (Fig. 4). The Egger's regression test (P = 0.002) revealed the presence of publication bias **(Appendix).** However, there is no change in the pooled prevalence after the trim and fill analysis. The QFT test was used in 29 studies where the largest and the smallest LTBI prevalence among CKD patients detected by QFT were 54% [39], and 6% [25], respectively. From 4821 CKD patients screened for LTBI using QFT, 1285 were found to have LTBI that gave a pooled prevalence of 28.0% (95%CI; 23.9, 32.0, I^2;^ 90.83%) (Fig. 5). The presence of publication bias was revealed by the asymmetry of the funnel plot and the statistical significance of the Egger's regression test (P = 0.0003) **(Appendix)**. After the trim and fill analysis, the pooled estimate became 25.4% (95%CI; 21.1%, 29.8%). The other laboratory method used to detect LTBI in CKD patients was T-SPOT that is used by 13 studies. Based on this method, the smallest and the highest LTBI prevalence was found to be 4% [25] and 58% [40], respectively. Among 1412 CKD patients screened for LTBI using T-SPOT, 595 patients were found to have LTBI. Based on the random effect model, the pooled prevalence of LTBI in CKD patients screened by T-SPOT was 32.6% (95% CI; 23.7, 41.5, I^2^; 97.53%) (Fig. 6). Based on the Egger's regression test there is no publication bias (P = 0.306) **(Appendix).** Since there is only one study that used ELISPOT, it was difficult to estimate the pooled prevalence **(**Table 2**).**

### 3.4 Prevalence of latent tuberculosis across categories of chronic kidney disease

**Fig. 7:**
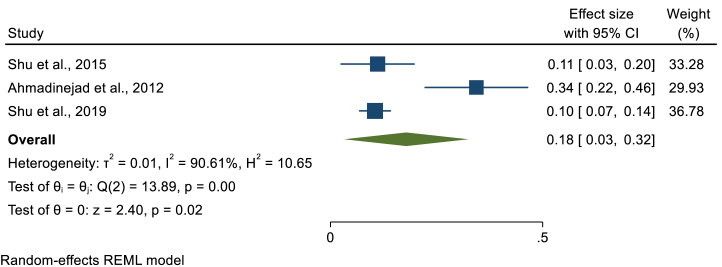
Forest plot for the pooled prevalence of latent tuberculosis among pre-dialysis chronic kidney disease patients.

**Fig. 8:**
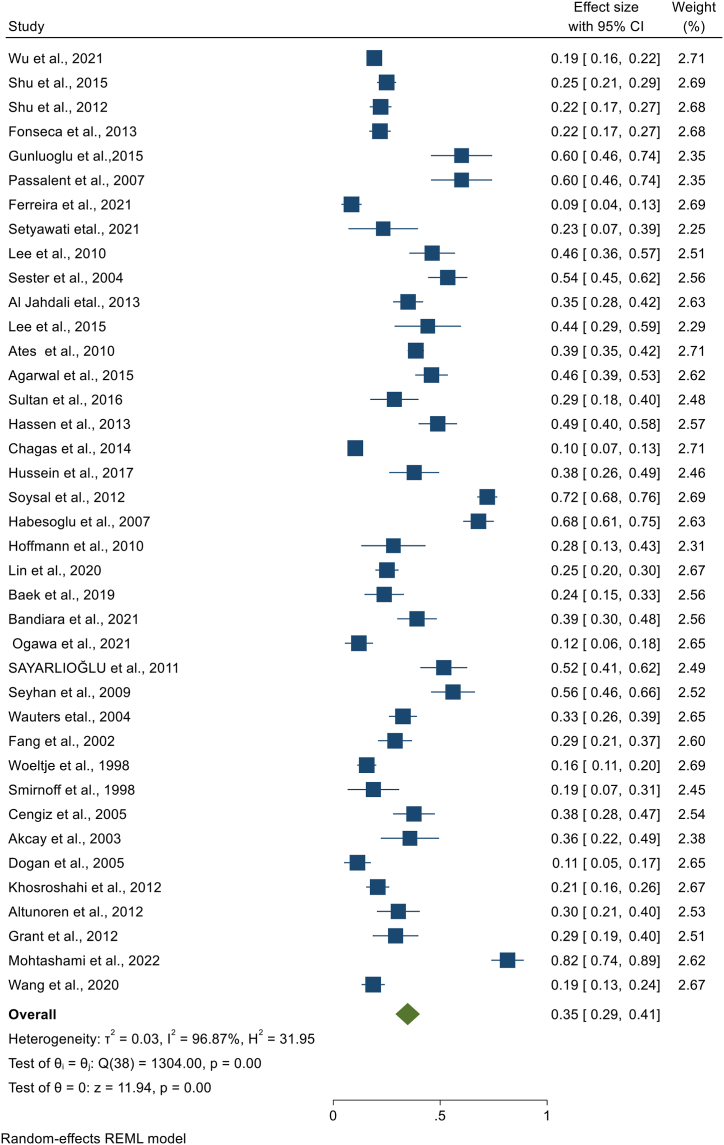
Forest plot for the pooled prevalence of latent tuberculosis among hemodialysis patients.

**Fig. 9:**
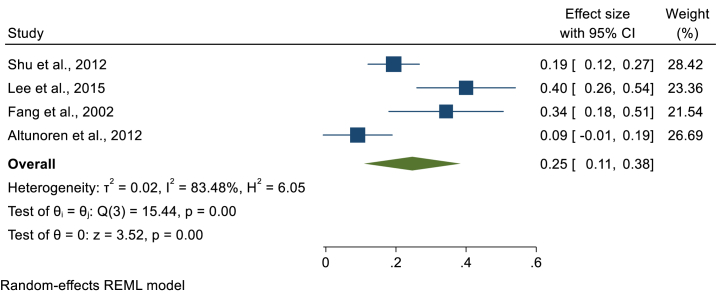
Forest plot for the pooled prevalence of latent tuberculosis among peritoneal dialysis patients.

**Fig. 10:**
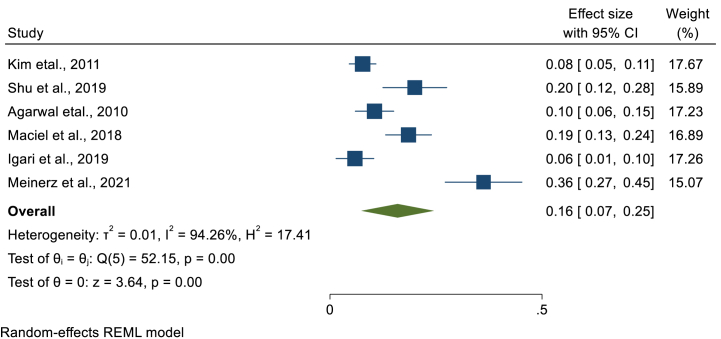
Forest plot for the pooled prevalence of latent tuberculosis among patients who undergone renal transplantation.

In this study, we have performed a sub-group analysis to estimate the pooled prevalence of LTBI based on the category of CKD such that pre-dialysis, hemodialysis, peritoneal dialysis and post-renal transplantation. Specifically, 39, 6, 5, and 3 studies assessed the prevalence of LTBI in hemodialysis, post-renal transplantation, peritoneal dialysis and pre-dialysis patients, respectively. Among 432 pre-dialysis patients, 61 were found to have LTBI that gave a pooled LTBI prevalence of 17.8% (95%CI; 3.3, 32.4, I^2^; 90.61%) (Fig. 7). In hemodialysis patients, the smallest prevalence of LTBI was 9% [12], while the highest prevalence was 82% [23]. A total of 7534 hemodialysis patients were screened for LTBI and 2396 were found to have LTBI with a pooled LTBI prevalence of 34.8% (95%CI; 29.1, 40.5, I^2^; 96.87%) (Fig. 8). The Egger's regression test was on the borderline (P = 0.049) that revealed the presence of publication bias. However, after the trim and fill analysis, there was no change in the pooled estimate. The third group of patients were those on peritoneal dialysis. We have estimated the pooled prevalence using four studies having 60 LTBI cases among 253 patients that gives a pooled prevalence of 25% (95%CI; 11, 38, I^2^; 83.48%) (Fig. 9). The last group of CKD patients were those who had undergone renal transplantation. The pooled prevalence of LTBI among CKD patients who underwent transplantation was estimated using six studies that comprises 1100 renal transplanted patients. LTBI was detected in 159 patients that gave a pooled LTBI prevalence of 16% (95%CI; 7, 25, I^2^; 94.26%) (Fig. 10) **(**Table 2**).**

We conducted a meta-regression analysis to assess the effect of sample size and publication year on the heterogeneity among studies. The multivariate meta-regression model revealed that sample size (P = 0.064) and publication year (0.553) did not significantly predicted heterogeneity among studies. However, this model only explains 4.58% of the heterogeneity **(**Table 3**).**

**Table 3:** Meta-regression analysis of heterogeneity using sample size and publication year.

Variable	Unadjusted model		Adjusted model	
	Coefficient (95%CI)	P-value	Coefficient (95%CI)	P-value
Sample size	−.0001712 (-.0003371 -5.31e-06)	0.043	−.0001609 (−.0003313 9.56e-06)	0.064
Publication year	−.0037576 (−.0114016, .0038865)	0.335	−.002313 (−.0099455 .0053196)	0.553

## 4 Discussion

In this study, we estimated the pooled prevalence of latent tuberculosis among patients with chronic kidney disease based on data collected from 53 studies that included 12,772 CKD patients. The study findings indicated that nearly one-third of CKD patients had LTBI with regional disparities. In addition, we conducted a sub-group analysis to estimate the pooled prevalence of LTBI based on the type of CKD, the laboratory diagnostic methods, continent, WHO regional classification, country's income level, publication year, and TB burden classification.

This study revealed that, 30% of CKD patients had LTBI, which is higher compared to the prevalence in the general population, where one-fourth of the global population is infected with TB [1]. In addition, this pooled estimate exceeds the global pooled estimate obtained among the general population, which was less than 25% [41]. This higher LTBI prevalence among CKD patients indicated that this group of population are at higher risk to develop active TB. This was corroborated in our recent meta-analysis, in which 3718/100, 000 CKD patients got TB during their follow-up period, substantially above the TB incidence in the general population [4]. This emphasizes the necessity of early and active screening, testing and treatment of LTBI in CKD patients in order to strengthen active TB prevention and control, which can improve the quality of life in this population. The outcomes of this study can be used to develop future guidelines and guidance. According to the WHO regional classification of countries, the highest pooled estimate was found in EUR (43%), followed by EMR (39%), SEAR (32%), WPR (21%), and AMR (19%). In a previous global meta-analysis study the decreasing order of the pooled prevalence of LTBI among the general population stratified per WHO regional classification was SEAR, AFR, EMR, WPR, AMR and EUR. Since we did not get studies from the WHO African region, we were unable to find the estimate the pooled estimate.

We also conducted a sub-group analysis based on publication year, and the study findings revealed that the pooled estimate is lower in studies published after 2016 (24%) compared to studies published between 1998 and 2010 (34%), and between 2011 and 2015 (34%). A global study [42] similarly found a modest reduction in the prevalence of LTBI. In addition, we have also estimated the pooled prevalence based on country's TB burden classification. The findings revealed that countries not included in the high TB burden countries had a relatively greater prevalence than their counterparts did.

The current study found that dialysis patients in general and hemodialysis patients in particular, had higher LTBI prevalence as compared to pre-dialysis and post-renal transplanted patients. Dialysis patients, particularly those on hemodialysis, are at increased risk of contracting *Mycobacterium tuberculosis* through person-to-person transmission since they travel frequently and spend lengthy periods in health facilities.

Besides, we have performed a sub-group analysis based on the laboratory diagnostic method used to diagnose LTBI in CKD patients. We found a relatively a higher pooled estimate in CKD patients diagnosed with IGRAs compared with TST. One possible reason might be the use of 10 mm cut-off in the TST. Likewise, this was reported by a previous global pooled estimate conducted in the general population [31].

In general, CKD patients suffer from multitude complications ranging from anemia, psychiatric diseases, cardiovascular complications, endocrine and metabolic abnormalities that needs to be given a focus to decrease high morbidity, mortality and poor quality of life [[43], [44], [45]].

Finally, the findings of this study should be interpreted by considering the following limitations. Primarily, under representation of CDKs from Africa in this review may have affected the global prevalence of LTBI in CDK patients. Second, the high heterogeneity among studies and the presence of publication bias may affect the true estimates. Lastly, since most of the original studies did not use specific cutoffs based on age and immunosuppression status of CKD patients, we did not perform analysis based on the specific cutoffs. However, we have performed stratified analysis that validated the current study findings.

## 5 Conclusion

This study identified higher prevalence of LTBI among CKD patients that needs attention of all concerned bodies to early detect and treat LTBI in this group of individual. There is disparities in the prevalence of LTBI per WHO regional classification, where CKD patients residing in the EUR, EMR and SEAR had relatively higher LTBI prevalence. In addition, dialysis patients mainly hemodialysis patients had higher LTBI prevalence compared to pre-dialysis and post-renal transplanted CKD patients. Besides, the prevalence of LTBI is higher in patients diagnosed with IGRA compared with those CKD patients diagnosed with TST. The findings in this study indicate the need to give attention for the early diagnosis and treatment of LTBI in CKD patients. We recommended more studies from the African Region where TB is endemic and the prevalence of CKD is increasing.

## Author contribution statement

Ayinalem Alemu: Conceived and designed the experiments; Performed the experiments; Analyzed and interpreted the data; Wrote the paper.

Zebenay Workneh Bitew: Performed the experiments; Analyzed and interpreted the data; Contributed reagents, materials, analysis tools or data.

Getu Diriba; Getachew Seid; Emebet Gashu: Performed the experiments.

Shewki Moga; Saro Abdella; Kirubel Eshetu; Getachew Tollera; Mesay Hailu Dangisso; Balako Gumi: Analyzed and interpreted the data; Wrote the paper.

## Data availability statement

Data included in article/supp. material/referenced in article.

## Declaration of competing interest

The authors declare that they have no known competing financial interests or personal relationships that could have appeared to influence the work reported in this paper.
