# Supplementary material for: The prevalence of latent tuberculosis infection in patients with chronic kidney disease: A systematic review and meta-analysis
Source: Heliyon. 2023 Jun 10;9(6):e17181. doi: 10.1016/j.heliyon.2023.e17181 (PMC10361307; doi:10.1016/j.heliyon.2023.e17181)
Supplement: Multimedia component 1 [file mmc1.docx]

**Appendix**

**Title: The prevalence of latent tuberculosis infection in patients with chronic kidney disease: a systematic review and meta-analysis**

Ayinalem Alemu^*1,2^, Zebenay Workneh Bitew^3^, Getu Diriba^1^, Getachew Seid^1,2^, Shewki Moga^1^, Saro Abdella^1^, Emebet Gashu^4^, Kirubel Eshetu^5^, Getachew Tolera^1^, Mesay Hailu Dangisso^1^, Balako Gumi^2^

^1^Ethiopian Public Health Institute, Addis Ababa, Ethiopia.

^2^Aklilu Lemma Institute of Pathobiology, Addis Ababa University, Addis Ababa, Ethiopia.

^3^St. Paul’s Hospital Millennium Medical College, Addis Ababa, Ethiopia.

^4^Addis Ababa Health Bureau, Addis Ababa, Ethiopia.

^5^USAID Eliminate TB Project, Management Sciences for Health, Addis Ababa, Ethiopia

# Table of contents

[Table of contents 2](#_Toc118208202)

[Search engines 3](#_Toc118208203)

[Completed PRISMA 2009 Checklist 7](#_Toc118208204)

[Quality assessment for the included studies in meta-analysis 9](#_Toc118208205)

[Supplementary figures 11](#_Toc118208206)

[Reference list of included studies 16](#_Toc118208207)

# Search engines

**PubMed**

| Search number | Query | | Sort By | | | Filters | Search Details | Results | Time |
| --- | --- | --- | --- | --- | --- | --- | --- | --- | --- |
| 16 | ((((("Latent Tuberculosis"[Mesh]) AND ("Kidney Failure, Chronic"[Mesh] OR "Renal Insufficiency"[Mesh] OR "Renal Failure, Progressive, with Hypertension" [Supplementary Concept])) OR (renal failure)) OR ("Kidney Transplantation"[Mesh])) OR ("Renal Dialysis"[Mesh] OR "Dialysis"[Mesh] OR "Peritoneal Dialysis, Continuous Ambulatory"[Mesh])) OR (hemodialysis) | Free full text, Full text, Observational Study, in the last 10 years, Humans, English, MEDLINE | | (("Latent Tuberculosis"[MeSH Terms] AND ("kidney failure, chronic"[MeSH Terms] OR "Renal Insufficiency"[MeSH Terms] OR "renal failure progressive with hypertension"[Supplementary Concept])) OR ("Renal Insufficiency"[MeSH Terms] OR ("renal"[All Fields] AND "insufficiency"[All Fields]) OR "Renal Insufficiency"[All Fields] OR ("renal"[All Fields] AND "failure"[All Fields]) OR "renal failure"[All Fields]) OR "Kidney Transplantation"[MeSH Terms] OR ("Renal Dialysis"[MeSH Terms] OR "Dialysis"[MeSH Terms] OR "peritoneal dialysis, continuous ambulatory"[MeSH Terms]) OR ("haemodialysis"[All Fields] OR "Renal Dialysis"[MeSH Terms] OR ("renal"[All Fields] AND "Dialysis"[All Fields]) OR "Renal Dialysis"[All Fields] OR "hemodialysis"[All Fields])) AND ((y_10[Filter]) AND (ffrft[Filter]) AND (observationalstudy[Filter]) AND (fft[Filter]) AND (humans[Filter]) AND (medline[Filter]) AND (english[Filter])) | | | | 3,914 | 4:35:20 |
| 15 | ((((("Latent Tuberculosis"[Mesh]) AND ("Kidney Failure, Chronic"[Mesh] OR "Renal Insufficiency"[Mesh] OR "Renal Failure, Progressive, with Hypertension" [Supplementary Concept])) OR (renal failure)) OR ("Kidney Transplantation"[Mesh])) OR ("Renal Dialysis"[Mesh] OR "Dialysis"[Mesh] OR "Peritoneal Dialysis, Continuous Ambulatory"[Mesh])) OR (hemodialysis) | Full text, Observational Study, in the last 10 years, Humans, English, MEDLINE | | (("Latent Tuberculosis"[MeSH Terms] AND ("kidney failure, chronic"[MeSH Terms] OR "Renal Insufficiency"[MeSH Terms] OR "renal failure progressive with hypertension"[Supplementary Concept])) OR ("Renal Insufficiency"[MeSH Terms] OR ("renal"[All Fields] AND "insufficiency"[All Fields]) OR "Renal Insufficiency"[All Fields] OR ("renal"[All Fields] AND "failure"[All Fields]) OR "renal failure"[All Fields]) OR "Kidney Transplantation"[MeSH Terms] OR ("Renal Dialysis"[MeSH Terms] OR "Dialysis"[MeSH Terms] OR "peritoneal dialysis, continuous ambulatory"[MeSH Terms]) OR ("haemodialysis"[All Fields] OR "Renal Dialysis"[MeSH Terms] OR ("renal"[All Fields] AND "Dialysis"[All Fields]) OR "Renal Dialysis"[All Fields] OR "hemodialysis"[All Fields])) AND ((y_10[Filter]) AND (observationalstudy[Filter]) AND (fft[Filter]) AND (humans[Filter]) AND (medline[Filter]) AND (english[Filter])) | | | | 7,288 | 4:35:11 |
| 14 | ((((("Latent Tuberculosis"[Mesh]) AND ("Kidney Failure, Chronic"[Mesh] OR "Renal Insufficiency"[Mesh] OR "Renal Failure, Progressive, with Hypertension" [Supplementary Concept])) OR (renal failure)) OR ("Kidney Transplantation"[Mesh])) OR ("Renal Dialysis"[Mesh] OR "Dialysis"[Mesh] OR "Peritoneal Dialysis, Continuous Ambulatory"[Mesh])) OR (hemodialysis) | Observational Study, in the last 10 years, Humans, English, MEDLINE | | (("Latent Tuberculosis"[MeSH Terms] AND ("kidney failure, chronic"[MeSH Terms] OR "Renal Insufficiency"[MeSH Terms] OR "renal failure progressive with hypertension"[Supplementary Concept])) OR ("Renal Insufficiency"[MeSH Terms] OR ("renal"[All Fields] AND "insufficiency"[All Fields]) OR "Renal Insufficiency"[All Fields] OR ("renal"[All Fields] AND "failure"[All Fields]) OR "renal failure"[All Fields]) OR "Kidney Transplantation"[MeSH Terms] OR ("Renal Dialysis"[MeSH Terms] OR "Dialysis"[MeSH Terms] OR "peritoneal dialysis, continuous ambulatory"[MeSH Terms]) OR ("haemodialysis"[All Fields] OR "Renal Dialysis"[MeSH Terms] OR ("renal"[All Fields] AND "Dialysis"[All Fields]) OR "Renal Dialysis"[All Fields] OR "hemodialysis"[All Fields])) AND ((y_10[Filter]) AND (observationalstudy[Filter]) AND (humans[Filter]) AND (medline[Filter]) AND (english[Filter])) | | | | 7,415 | 4:34:51 |
| 13 | ((((("Latent Tuberculosis"[Mesh]) AND ("Kidney Failure, Chronic"[Mesh] OR "Renal Insufficiency"[Mesh] OR "Renal Failure, Progressive, with Hypertension" [Supplementary Concept])) OR (renal failure)) OR ("Kidney Transplantation"[Mesh])) OR ("Renal Dialysis"[Mesh] OR "Dialysis"[Mesh] OR "Peritoneal Dialysis, Continuous Ambulatory"[Mesh])) OR (hemodialysis) | Observational Study, in the last 10 years, Humans, English | | (("Latent Tuberculosis"[MeSH Terms] AND ("kidney failure, chronic"[MeSH Terms] OR "Renal Insufficiency"[MeSH Terms] OR "renal failure progressive with hypertension"[Supplementary Concept])) OR ("Renal Insufficiency"[MeSH Terms] OR ("renal"[All Fields] AND "insufficiency"[All Fields]) OR "Renal Insufficiency"[All Fields] OR ("renal"[All Fields] AND "failure"[All Fields]) OR "renal failure"[All Fields]) OR "Kidney Transplantation"[MeSH Terms] OR ("Renal Dialysis"[MeSH Terms] OR "Dialysis"[MeSH Terms] OR "peritoneal dialysis, continuous ambulatory"[MeSH Terms]) OR ("haemodialysis"[All Fields] OR "Renal Dialysis"[MeSH Terms] OR ("renal"[All Fields] AND "Dialysis"[All Fields]) OR "Renal Dialysis"[All Fields] OR "hemodialysis"[All Fields])) AND ((y_10[Filter]) AND (observationalstudy[Filter]) AND (humans[Filter]) AND (english[Filter])) | | | | 7,415 | 4:34:34 |
| 12 | ((((("Latent Tuberculosis"[Mesh]) AND ("Kidney Failure, Chronic"[Mesh] OR "Renal Insufficiency"[Mesh] OR "Renal Failure, Progressive, with Hypertension" [Supplementary Concept])) OR (renal failure)) OR ("Kidney Transplantation"[Mesh])) OR ("Renal Dialysis"[Mesh] OR "Dialysis"[Mesh] OR "Peritoneal Dialysis, Continuous Ambulatory"[Mesh])) OR (hemodialysis) | Observational Study, Humans, English | | (("Latent Tuberculosis"[MeSH Terms] AND ("kidney failure, chronic"[MeSH Terms] OR "Renal Insufficiency"[MeSH Terms] OR "renal failure progressive with hypertension"[Supplementary Concept])) OR ("Renal Insufficiency"[MeSH Terms] OR ("renal"[All Fields] AND "insufficiency"[All Fields]) OR "Renal Insufficiency"[All Fields] OR ("renal"[All Fields] AND "failure"[All Fields]) OR "renal failure"[All Fields]) OR "Kidney Transplantation"[MeSH Terms] OR ("Renal Dialysis"[MeSH Terms] OR "Dialysis"[MeSH Terms] OR "peritoneal dialysis, continuous ambulatory"[MeSH Terms]) OR ("haemodialysis"[All Fields] OR "Renal Dialysis"[MeSH Terms] OR ("renal"[All Fields] AND "Dialysis"[All Fields]) OR "Renal Dialysis"[All Fields] OR "hemodialysis"[All Fields])) AND ((observationalstudy[Filter]) AND (humans[Filter]) AND (english[Filter])) | | | | 7,428 | 4:34:22 |
| 11 | ((((("Latent Tuberculosis"[Mesh]) AND ("Kidney Failure, Chronic"[Mesh] OR "Renal Insufficiency"[Mesh] OR "Renal Failure, Progressive, with Hypertension" [Supplementary Concept])) OR (renal failure)) OR ("Kidney Transplantation"[Mesh])) OR ("Renal Dialysis"[Mesh] OR "Dialysis"[Mesh] OR "Peritoneal Dialysis, Continuous Ambulatory"[Mesh])) OR (hemodialysis) | Observational Study, Humans | | (("Latent Tuberculosis"[MeSH Terms] AND ("kidney failure, chronic"[MeSH Terms] OR "Renal Insufficiency"[MeSH Terms] OR "renal failure progressive with hypertension"[Supplementary Concept])) OR ("Renal Insufficiency"[MeSH Terms] OR ("renal"[All Fields] AND "insufficiency"[All Fields]) OR "Renal Insufficiency"[All Fields] OR ("renal"[All Fields] AND "failure"[All Fields]) OR "renal failure"[All Fields]) OR "Kidney Transplantation"[MeSH Terms] OR ("Renal Dialysis"[MeSH Terms] OR "Dialysis"[MeSH Terms] OR "peritoneal dialysis, continuous ambulatory"[MeSH Terms]) OR ("haemodialysis"[All Fields] OR "Renal Dialysis"[MeSH Terms] OR ("renal"[All Fields] AND "Dialysis"[All Fields]) OR "Renal Dialysis"[All Fields] OR "hemodialysis"[All Fields])) AND ((observationalstudy[Filter]) AND (humans[Filter])) | | | | 7,586 | 4:34:15 |
| 10 | ((((("Latent Tuberculosis"[Mesh]) AND ("Kidney Failure, Chronic"[Mesh] OR "Renal Insufficiency"[Mesh] OR "Renal Failure, Progressive, with Hypertension" [Supplementary Concept])) OR (renal failure)) OR ("Kidney Transplantation"[Mesh])) OR ("Renal Dialysis"[Mesh] OR "Dialysis"[Mesh] OR "Peritoneal Dialysis, Continuous Ambulatory"[Mesh])) OR (hemodialysis) | Observational Study | | (("Latent Tuberculosis"[MeSH Terms] AND ("kidney failure, chronic"[MeSH Terms] OR "Renal Insufficiency"[MeSH Terms] OR "renal failure progressive with hypertension"[Supplementary Concept])) OR ("Renal Insufficiency"[MeSH Terms] OR ("renal"[All Fields] AND "insufficiency"[All Fields]) OR "Renal Insufficiency"[All Fields] OR ("renal"[All Fields] AND "failure"[All Fields]) OR "renal failure"[All Fields]) OR "Kidney Transplantation"[MeSH Terms] OR ("Renal Dialysis"[MeSH Terms] OR "Dialysis"[MeSH Terms] OR "peritoneal dialysis, continuous ambulatory"[MeSH Terms]) OR ("haemodialysis"[All Fields] OR "Renal Dialysis"[MeSH Terms] OR ("renal"[All Fields] AND "Dialysis"[All Fields]) OR "Renal Dialysis"[All Fields] OR "hemodialysis"[All Fields])) AND (observationalstudy[Filter]) | | | | 7,594 | 4:33:57 |
| 7 | ((((("Latent Tuberculosis"[Mesh]) AND ("Kidney Failure, Chronic"[Mesh] OR "Renal Insufficiency"[Mesh] OR "Renal Failure, Progressive, with Hypertension" [Supplementary Concept])) OR (renal failure)) OR ("Kidney Transplantation"[Mesh])) OR ("Renal Dialysis"[Mesh] OR "Dialysis"[Mesh] OR "Peritoneal Dialysis, Continuous Ambulatory"[Mesh])) OR (hemodialysis) | | | ("Latent Tuberculosis"[MeSH Terms] AND ("kidney failure, chronic"[MeSH Terms] OR "Renal Insufficiency"[MeSH Terms] OR "renal failure progressive with hypertension"[Supplementary Concept])) OR ("Renal Insufficiency"[MeSH Terms] OR ("renal"[All Fields] AND "insufficiency"[All Fields]) OR "Renal Insufficiency"[All Fields] OR ("renal"[All Fields] AND "failure"[All Fields]) OR "renal failure"[All Fields]) OR "Kidney Transplantation"[MeSH Terms] OR ("Renal Dialysis"[MeSH Terms] OR "Dialysis"[MeSH Terms] OR "peritoneal dialysis, continuous ambulatory"[MeSH Terms]) OR ("haemodialysis"[All Fields] OR "Renal Dialysis"[MeSH Terms] OR ("renal"[All Fields] AND "Dialysis"[All Fields]) OR "Renal Dialysis"[All Fields] OR "hemodialysis"[All Fields]) | | | | 461,481 | 4:33:48 |
| 9 | ((((("Latent Tuberculosis"[Mesh]) AND ("Kidney Failure, Chronic"[Mesh] OR "Renal Insufficiency"[Mesh] OR "Renal Failure, Progressive, with Hypertension" [Supplementary Concept])) OR (renal failure)) OR ("Kidney Transplantation"[Mesh])) OR ("Renal Dialysis"[Mesh] OR "Dialysis"[Mesh] OR "Peritoneal Dialysis, Continuous Ambulatory"[Mesh])) OR (hemodialysis) | Humans, English | | (("Latent Tuberculosis"[MeSH Terms] AND ("kidney failure, chronic"[MeSH Terms] OR "Renal Insufficiency"[MeSH Terms] OR "renal failure progressive with hypertension"[Supplementary Concept])) OR ("Renal Insufficiency"[MeSH Terms] OR ("renal"[All Fields] AND "insufficiency"[All Fields]) OR "Renal Insufficiency"[All Fields] OR ("renal"[All Fields] AND "failure"[All Fields]) OR "renal failure"[All Fields]) OR "Kidney Transplantation"[MeSH Terms] OR ("Renal Dialysis"[MeSH Terms] OR "Dialysis"[MeSH Terms] OR "peritoneal dialysis, continuous ambulatory"[MeSH Terms]) OR ("haemodialysis"[All Fields] OR "Renal Dialysis"[MeSH Terms] OR ("renal"[All Fields] AND "Dialysis"[All Fields]) OR "Renal Dialysis"[All Fields] OR "hemodialysis"[All Fields])) AND ((humans[Filter]) AND (english[Filter])) | | | | 338,560 | 4:32:20 |
| 8 | ((((("Latent Tuberculosis"[Mesh]) AND ("Kidney Failure, Chronic"[Mesh] OR "Renal Insufficiency"[Mesh] OR "Renal Failure, Progressive, with Hypertension" [Supplementary Concept])) OR (renal failure)) OR ("Kidney Transplantation"[Mesh])) OR ("Renal Dialysis"[Mesh] OR "Dialysis"[Mesh] OR "Peritoneal Dialysis, Continuous Ambulatory"[Mesh])) OR (hemodialysis) | Humans | | (("Latent Tuberculosis"[MeSH Terms] AND ("kidney failure, chronic"[MeSH Terms] OR "Renal Insufficiency"[MeSH Terms] OR "renal failure progressive with hypertension"[Supplementary Concept])) OR ("Renal Insufficiency"[MeSH Terms] OR ("renal"[All Fields] AND "insufficiency"[All Fields]) OR "Renal Insufficiency"[All Fields] OR ("renal"[All Fields] AND "failure"[All Fields]) OR "renal failure"[All Fields]) OR "Kidney Transplantation"[MeSH Terms] OR ("Renal Dialysis"[MeSH Terms] OR "Dialysis"[MeSH Terms] OR "peritoneal dialysis, continuous ambulatory"[MeSH Terms]) OR ("haemodialysis"[All Fields] OR "Renal Dialysis"[MeSH Terms] OR ("renal"[All Fields] AND "Dialysis"[All Fields]) OR "Renal Dialysis"[All Fields] OR "hemodialysis"[All Fields])) AND (humans[Filter]) | | | | 391,999 | 4:32:13 |
| 6 | hemodialysis |  | | "haemodialysis"[All Fields] OR "renal dialysis"[MeSH Terms] OR ("renal"[All Fields] AND "dialysis"[All Fields]) OR "renal dialysis"[All Fields] OR "hemodialysis"[All Fields] | | | | 171,266 | 4:29:58 |
| 5 | renal failure |  | | "renal insufficiency"[MeSH Terms] OR ("renal"[All Fields] AND "insufficiency"[All Fields]) OR "renal insufficiency"[All Fields] OR ("renal"[All Fields] AND "failure"[All Fields]) OR "renal failure"[All Fields] | | | | 289,072 | 4:28:42 |
| 4 | "Kidney Failure, Chronic"[Mesh] OR "Renal Insufficiency"[Mesh] OR "Renal Failure, Progressive, with Hypertension" [Supplementary Concept] | | Most Recent | | "kidney failure, chronic"[MeSH Terms] OR "Renal Insufficiency"[MeSH Terms] OR "renal failure progressive with hypertension"[Supplementary Concept] | | | 196,663 | 4:28:14 |
| 3 | "Kidney Transplantation"[Mesh] | | Most Recent | | "Kidney Transplantation"[MeSH Terms] | | | 103,365 | 4:26:45 |
| 2 | "Renal Dialysis"[Mesh] OR "Dialysis"[Mesh] OR "Peritoneal Dialysis, Continuous Ambulatory"[Mesh] | | Most Recent | | "Renal Dialysis"[MeSH Terms] OR "Dialysis"[MeSH Terms] OR "peritoneal dialysis, continuous ambulatory"[MeSH Terms] | | | 146,512 | 4:24:43 |
| 1 | "Latent Tuberculosis"[Mesh] | | Most Recent | | "Latent Tuberculosis"[MeSH Terms] | | | 3,689 | 4:22:40 |

**Global Index Medicus**


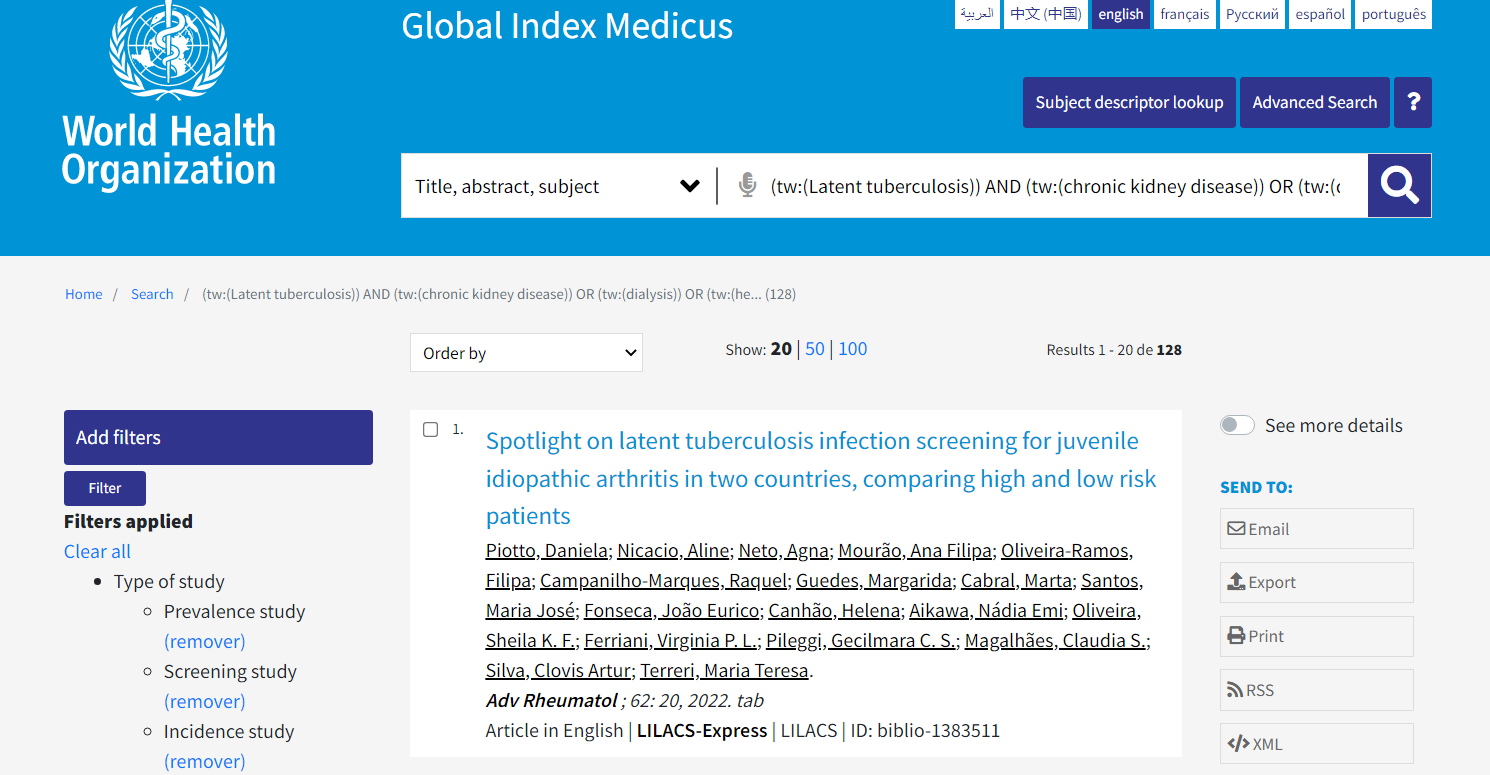


**Informit**


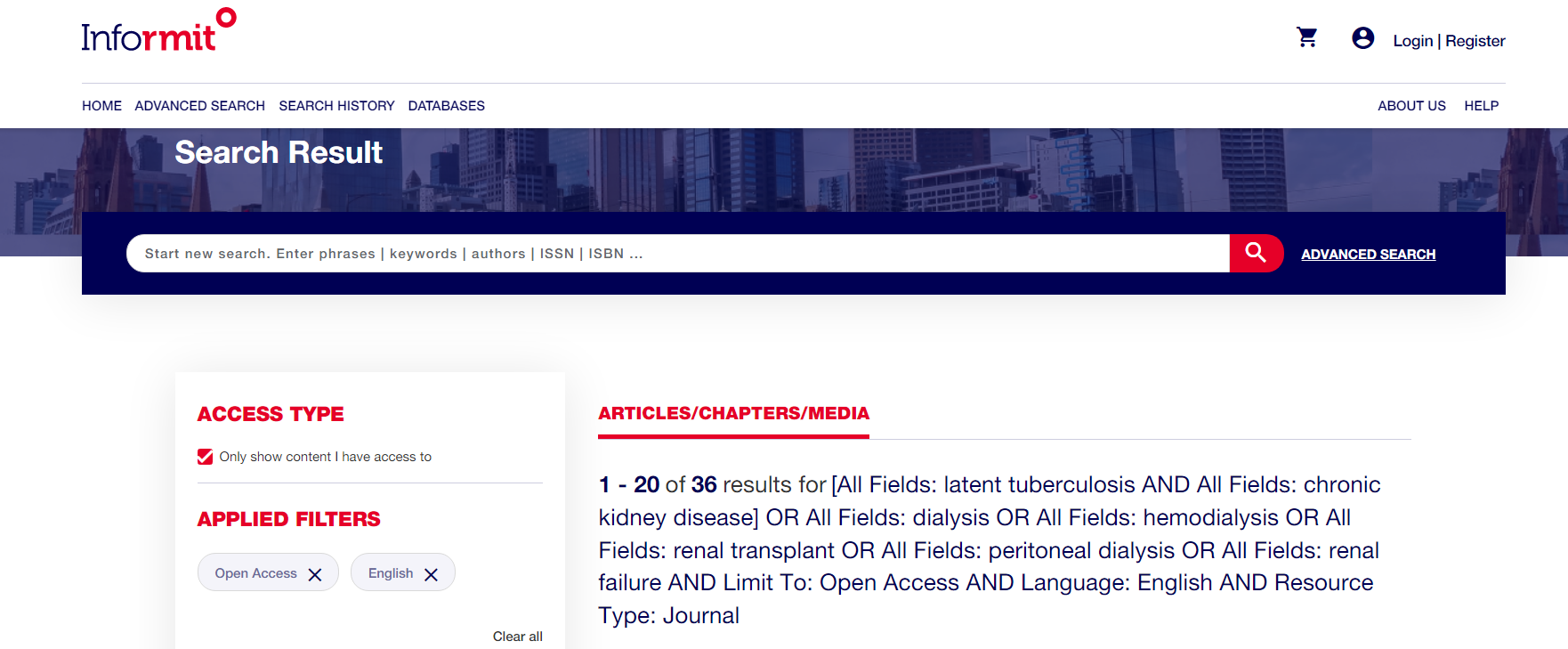


**Joanna Briggs Institute EBP Database (including OVID)**


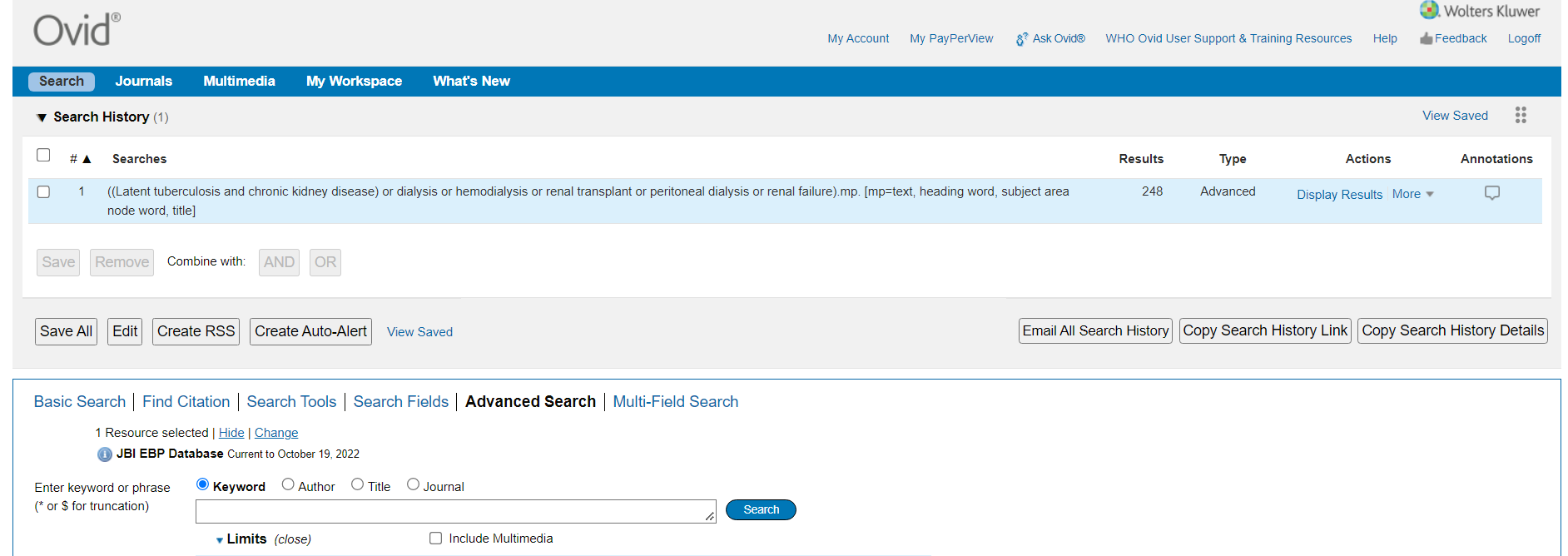


**Global Health/CABI**


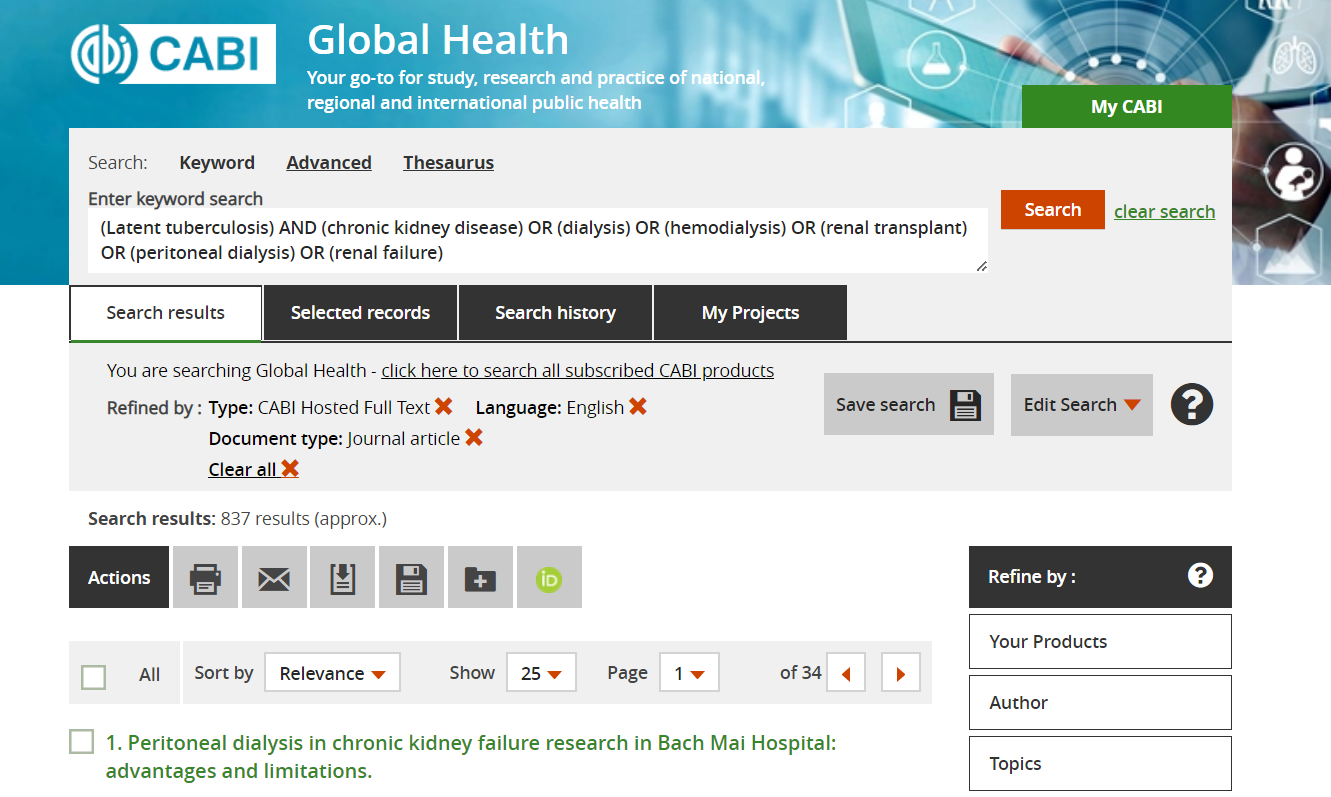


#
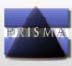
Completed PRISMA 2009 Checklist

| **Section/topic** | **#** | **Checklist item** | **Reported on page #** |
| --- | --- | --- | --- |
| **TITLE** | | |  |
| Title | 1 | Identify the report as a systematic review, meta-analysis, or both. | 1 |
| **ABSTRACT** | | |  |
| Structured summary | 2 | Provide a structured summary including, as applicable: background; objectives; data sources; study eligibility criteria, participants, and interventions; study appraisal and synthesis methods; results; limitations; conclusions and implications of key findings; systematic review registration number. | 2 |
| **INTRODUCTION** | | |  |
| Rationale | 3 | Describe the rationale for the review in the context of what is already known. | 3-4 |
| Objectives | 4 | Provide an explicit statement of questions being addressed with reference to participants, interventions, comparisons, outcomes, and study design (PICOS). | 4 |
| **METHODS** | | |  |
| Protocol and registration | 5 | Indicate if a review protocol exists, if and where it can be accessed (e.g., Web address), and, if available, provide registration information including registration number. | - |
| Eligibility criteria | 6 | Specify study characteristics (e.g., PICOS, length of follow-up) and report characteristics (e.g., years considered, language, publication status) used as criteria for eligibility, giving rationale. | 5 |
| Information sources | 7 | Describe all information sources (e.g., databases with dates of coverage, contact with study authors to identify additional studies) in the search and date last searched. | 4-5 |
| Search | 8 | Present full electronic search strategy for at least one database, including any limits used, such that it could be repeated. | 5 |
| Study selection | 9 | State the process for selecting studies (i.e., screening, eligibility, included in systematic review, and, if applicable, included in the meta-analysis). | 4 |
| Data collection process | 10 | Describe method of data extraction from reports (e.g., piloted forms, independently, in duplicate) and any processes for obtaining and confirming data from investigators. | 6 |
| Data items | 11 | List and define all variables for which data were sought (e.g., PICOS, funding sources) and any assumptions and simplifications made. | 5 |
| Risk of bias in individual studies | 12 | Describe methods used for assessing risk of bias of individual studies (including specification of whether this was done at the study or outcome level), and how this information is to be used in any data synthesis. | 7 |
| Summary measures | 13 | State the principal summary measures (e.g., risk ratio, difference in means). | 7 |
| Synthesis of results | 14 | Describe the methods of handling data and combining results of studies, if done, including measures of consistency (e.g., I^2^) for each meta-analysis. | 7 |

Page 1 of 2

| **Section/topic** | **#** | **Checklist item** | **Reported on page #** |
| --- | --- | --- | --- |
| Risk of bias across studies | 15 | Specify any assessment of risk of bias that may affect the cumulative evidence (e.g., publication bias, selective reporting within studies). | 7 |
| Additional analyses | 16 | Describe methods of additional analyses (e.g., sensitivity or subgroup analyses, meta-regression), if done, indicating which were pre-specified. | 7 |
| **RESULTS** | | |  |
| Study selection | 17 | Give numbers of studies screened, assessed for eligibility, and included in the review, with reasons for exclusions at each stage, ideally with a flow diagram. | 8 |
| Study characteristics | 18 | For each study, present characteristics for which data were extracted (e.g., study size, PICOS, follow-up period) and provide the citations. | 8-9 |
| Risk of bias within studies | 19 | Present data on risk of bias of each study and, if available, any outcome level assessment (see item 12). | 9-12 |
| Results of individual studies | 20 | For all outcomes considered (benefits or harms), present, for each study: (a) simple summary data for each intervention group (b) effect estimates and confidence intervals, ideally with a forest plot. | 9-12 |
| Synthesis of results | 21 | Present results of each meta-analysis done, including confidence intervals and measures of consistency. | 9-12 |
| Risk of bias across studies | 22 | Present results of any assessment of risk of bias across studies (see Item 15). | 9-12 |
| Additional analysis | 23 | Give results of additional analyses, if done (e.g., sensitivity or subgroup analyses, meta-regression [see Item 16]). | 12 |
| **DISCUSSION** | | |  |
| Summary of evidence | 24 | Summarize the main findings including the strength of evidence for each main outcome; consider their relevance to key groups (e.g., healthcare providers, users, and policy makers). | 12-14 |
| Limitations | 25 | Discuss limitations at study and outcome level (e.g., risk of bias), and at review-level (e.g., incomplete retrieval of identified research, reporting bias). | 14 |
| Conclusions | 26 | Provide a general interpretation of the results in the context of other evidence, and implications for future research. | 14-15 |
| **FUNDING** | | |  |
| Funding | 27 | Describe sources of funding for the systematic review and other support (e.g., supply of data); role of funders for the systematic review. | 15 |

*From:*  Moher D, Liberati A, Tetzlaff J, Altman DG, The PRISMA Group (2009). Preferred Reporting Items for Systematic Reviews and Meta-Analyses: The PRISMA Statement. PLoS Med 6(7): e1000097. doi:10.1371/journal.pmed1000097

For more information, visit: **www.prisma-statement.org**.

Page 2 of 2

# Quality assessment for the included studies in meta-analysis

| Author year | Q1 | Q2 | Q3 | Q4 | Q5 | Q6 | Q7 | Q8 | Q9 | Over all quality score |
| --- | --- | --- | --- | --- | --- | --- | --- | --- | --- | --- |
| Wu et al., 2021 | Y | Y | Y | Y | Y | Y | Y | Y | Y | High |
| Shu et al., 2015 | Y | Y | Y | Y | Y | Y | Y | Y | Y | High |
| Shu et al., 2012 | Y | Y | Y | Y | Y | Y | Y | Y | Y | High |
| Fonseca et al., 2013 | Y | Y | Y | Y | Y | Y | Y | Y | Y | High |
| Gunluoglu et al., 2015 | Y | Y | N | Y | Y | Y | Y | Y | Y | High |
| Passalent et al., 2007 | Y | Y | Y | Y | Y | Y | Y | Y | Y | High |
| Ferreira et al., 2021 | Y | Y | Y | Y | Y | Y | Y | Y | Y | High |
| Setyawati etal., 2021 | Y | Y | N | Y | Y | Y | Y | Y | Y | High |
| Lee et al., 2010 | Y | Y | N | Y | Y | Y | Y | Y | Y | High |
| Ahmadinejad et al., 2012 | Y | Y | N | Y | Y | Y | Y | Y | Y | High |
| Sester et al., 2004 | Y | Y | Y | Y | Y | Y | Y | Y | Y | High |
| Al Jahdali etal., 2013 | Y | Y | Y | Y | Y | Y | Y | Y | Y | High |
| Kim etal., 2011 | Y | Y | Y | Y | Y | Y | Y | Y | Y | High |
| Al Wakeel et al., 2015 | Y | Y | Y | Y | Y | Y | Y | Y | Y | High |
| Lee et al., 2015 | Y | Y | N | Y | Y | Y | Y | Y | Y | High |
| Ates et al., 2010 | Y | Y | Y | Y | Y | Y | Y | Y | Y | High |
| Chung et al., 2009 | Y | Y | Y | Y | Y | Y | Y | Y | Y | High |
| Shu et al., 2019 | Y | Y | Y | Y | Y | Y | Y | Y | Y | High |
| Agarwal et al., 2015 | Y | Y | Y | Y | Y | Y | Y | Y | Y | High |
| Sultan et al., 2016 | Y | Y | N | Y | Y | Y | Y | Y | Y | High |
| Hassen et al., 2013 | Y | Y | Y | Y | Y | Y | Y | Y | Y | High |
| Chagas et al., 2014 | Y | Y | Y | N | Y | Y | Y | Y | Y | High |
| Romanowski., 2020 | Y | Y | Y | N | Y | Y | Y | Y | Y | High |
| Hussein et al., 2017 | Y | Y | N | Y | Y | Y | Y | Y | Y | High |
| Shankar et al., 2005 | Y | Y | Y | Y | Y | Y | Y | Y | Y | High |
| Triverio et al., 2009 | Y | Y | Y | Y | Y | Y | Y | Y | Y | High |
| Soysal et al., 2012 | Y | Y | Y | Y | Y | Y | Y | Y | Y | High |
| Habesoglu et al., 2007 | Y | Y | Y | Y | Y | Y | Y | Y | Y | High |
| Hoffmann et al., 2010 | Y | Y | Y | Y | Y | Y | Y | Y | Y | High |
| Agarwal etal., 2010 | Y | Y | Y | Y | Y | Y | Y | Y | Y | High |
| Lee etal., 2009 | Y | Y | Y | Y | Y | Y | Y | Y | Y | High |
| Lin et al., 2020 | Y | Y | Y | Y | Y | Y | Y | Y | Y | High |
| Baek et al., 2019 | Y | Y | N | Y | Y | Y | Y | Y | Y | High |
| Bandiara et al., 2021 | Y | Y | Y | Y | Y | Y | Y | Y | Y | High |
| Ogawa et al., 2021 | Y | Y | Y | N | Y | Y | Y | Y | Y | High |
| SAYARLIOĞLU et al., 2011 | Y | Y | N | Y | Y | Y | Y | Y | Y | High |
| Carrazco-Ibarra et al., 2017 | Y | Y | Y | N | Y | Y | Y | Y | Y | High |
| Seyhan et al., 2009 | Y | Y | Y | Y | Y | Y | Y | Y | Y | High |
| Wauters etal., 2004 | Y | Y | Y | N | Y | Y | Y | Y | Y | High |
| Fang et al., 2002 | Y | Y | Y | Y | Y | Y | Y | Y | Y | High |
| Winthrop et al., 2008 | Y | Y | Y | Y | Y | Y | Y | Y | Y | High |
| Woeltje et al., 1998 | Y | Y | Y | Y | Y | Y | Y | Y | Y | High |
| Smirnoff et al., 1998 | Y | Y | N | Y | Y | Y | Y | Y | Y | High |
| Connel et al., 2010 | Y | Y | N | Y | Y | Y | Y | Y | Y | High |
| Cengiz et al., 2005 | Y | Y | Y | Y | Y | Y | Y | Y | Y | High |
| Akcay et al., 2003 | Y | Y | N | Y | Y | Y | Y | Y | Y | High |
| Dogan et al., 2005 | Y | Y | Y | Y | Y | Y | Y | Y | Y | High |
| Foster et al., 2016 | Y | Y | Y | N | Y | Y | Y | Y | Y | High |
| Khosroshahi et al., 2012 | Y | Y | Y | N | Y | Y | Y | Y | Y | High |
| Altunoren et al., 2012 | Y | Y | Y | Y | Y | Y | Y | Y | Y | High |
| Edathodu et al., 2016 | Y | Y | Y | Y | Y | Y | Y | Y | Y | High |
| Maciel et al., 2018 | Y | Y | Y | Y | Y | Y | Y | Y | Y | High |
| Igari et al., 2019 | Y | Y | Y | Y | Y | Y | Y | Y | Y | High |
| Meinerz et al., 2021 | Y | Y | Y | N | Y | Y | Y | Y | Y | High |
| Grant et al., 2012 | Y | Y | N | Y | Y | Y | Y | Y | Y | High |
| Chung et al., 2010 | Y | Y | N | Y | Y | Y | Y | Y | Y | High |
| Mohtashami et al., 2022 | Y | Y | Y | Y | Y | Y | Y | Y | Y | High |
| Wang et al., 2020 | Y | Y | Y | Y | Y | Y | Y | Y | Y | High |
| Harris et al.,2016 | Y | Y | Y | Y | Y | Y | Y | Y | Y | High |

Y; Yes, N; No

# Supplementary figures


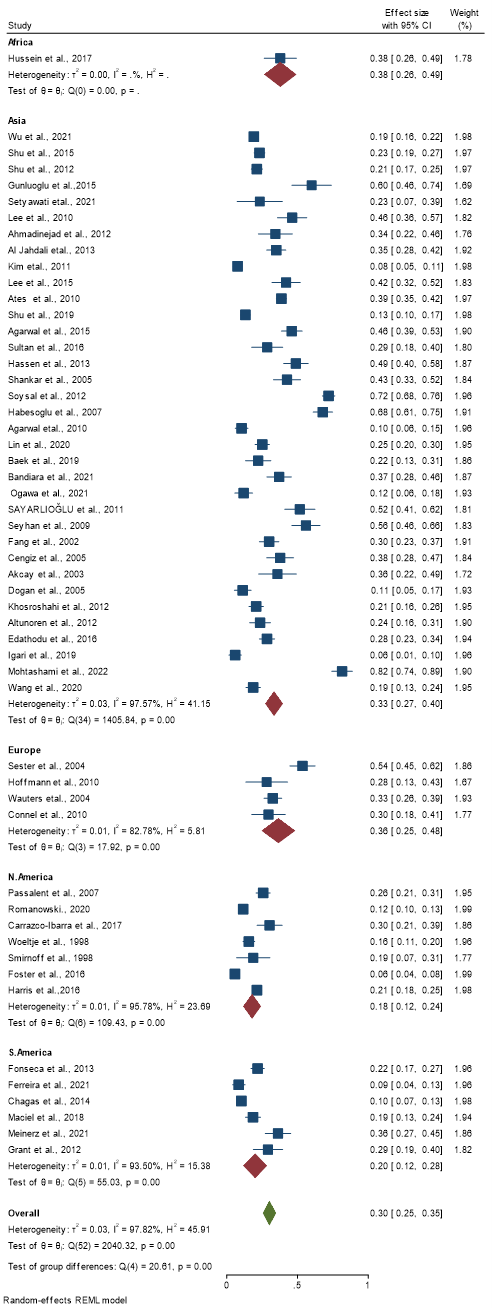


**Supplementary figure 1.** Forest plot for the pooled prevalence of latent tuberculosis among patients with chronic kidney disease diagnosed based on continent.


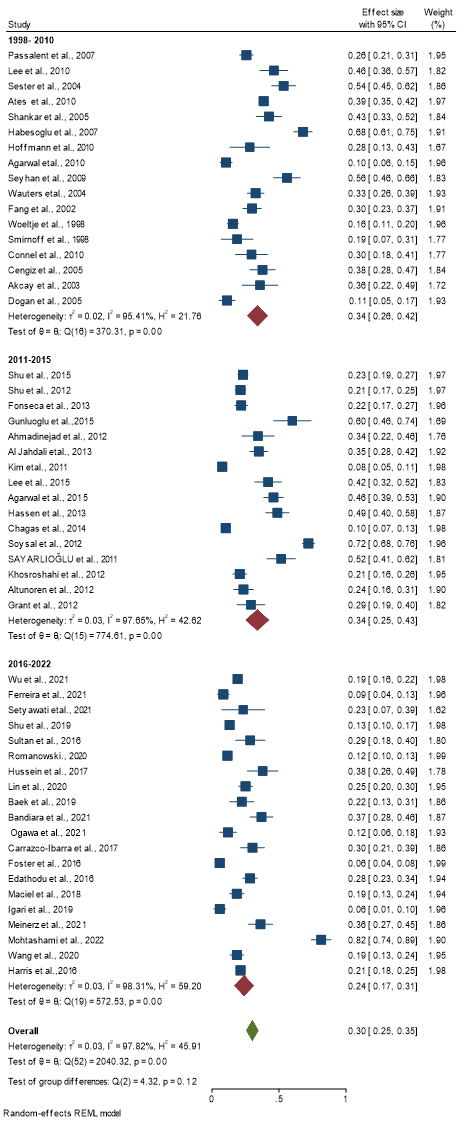


**Supplementary figure 2.** Forest plot for the pooled prevalence of latent tuberculosis among patients with chronic kidney disease diagnosed based on publication year.


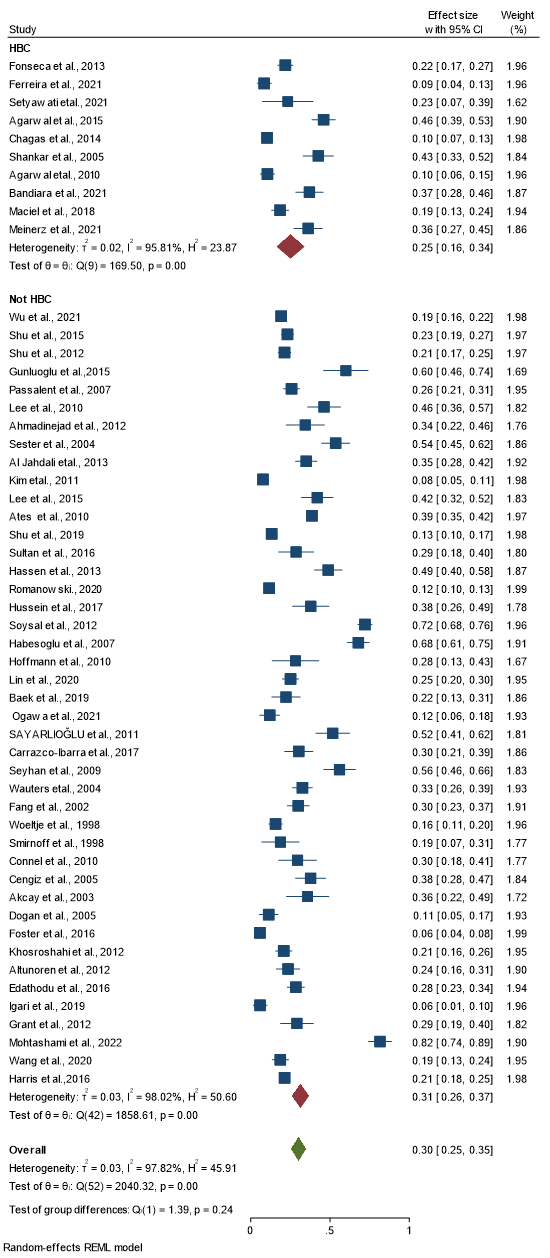


**Supplementary figure 3.** Forest plot for the pooled prevalence of latent tuberculosis among patients with chronic kidney disease diagnosed based on TB burden category of a country.

**Supplementary figure 4.** Forest plot for the pooled prevalence of latent tuberculosis among patients with chronic kidney disease diagnosed based on country’s income level.

**Supplementary figure 5.** Funnel plot for the pooled prevalence of latent tuberculosis among patients with chronic kidney disease diagnosed using TST.

**Supplementary figure 6.** Funnel plot for the pooled prevalence of latent tuberculosis among patients with chronic kidney disease diagnosed using QFT.

**Supplementary figure 7.** Funnel plot for the pooled prevalence of latent tuberculosis among patients with chronic kidney disease diagnosed using T-SPOT.

**Supplementary figure 78** Funnel plot for the pooled prevalence of latent tuberculosis among hemodialysis patients.

# Reference list of included studies

1. Wu CH, Su HA, Chou CA, Liu JW, Lee CT, Dai LH, et al. An observational study on prevalence of latent tuberculosis infection and outcome of 3HP treatment in patients under hemodialysis in Taiwan. Journal of the Formosan Medical Association = Taiwan yi zhi. 2021;120(6):1350-60.
2. Shu CC, Hsu CL, Lee CY, Wang JY, Wu VC, Yang FJ, et al. Comparison of the Prevalence of Latent Tuberculosis Infection among Non-Dialysis Patients with Severe Chronic Kidney Disease, Patients Receiving Dialysis, and the Dialysis-Unit Staff: A Cross-Sectional Study. PLoS One. 2015;10(4):e0124104.
3. Shu C-C, Wu V-C, Yang F-J, Pan S-C, Lai T-S, et al. Predictors and Prevalence of Latent Tuberculosis Infection in Patients Receiving Long-Term Hemodialysis and Peritoneal Dialysis. PLoS ONE. 2012; 7(8): e42592.
4. Fonseca JC, Caiaffa WT, Abreu MNS, Farah KP, Carvalho WS, et al. Prevalence of latent tuberculosis infection and risk of infection in patients with chronic kidney disease undergoing hemodialysis in a referral center in Brazil. J Bras Pneumol. 2013;39(2):214-220.
5. Gunluoglu G , Seyhan EC, Veske NS, Kazancioglu R, et al. Diagnosing Latent Tuberculosis in Immunocompromised Patients Measuring Blood IP-10 Production Capacity: An Analysis of Chronic Renal Failure Patients. Intern Med. 2015;54: 465-472
6. Passalent L, Khan K, Richardson R, Wang J, Dedier H and Gardam M. Detecting Latent Tuberculosis Infection in Hemodialysis Patients: A Head-to-Head Comparison of the T-SPOT.TB Test, Tuberculin Skin Test, and an Expert Physician Panel. Clin J Am Soc Nephrol. 2007; 2: 68–73.
7. Ferreira V, Fonseca CDD, Bollela VR, Romão EA, Costa J, Sousa AFL, et al. Prevalence of latent tuberculosis and associated factors in patients with chronic kidney disease on hemodialysis. Revista latino-americana de enfermagem. 2021;29:e3442.
8. Setyawati A, Reviono, Putranto W. THE COMPABILITY LEVEL OF TUBERCULIN SKIN TEST AND T-SPOT.TB, SENSITIVITY AND SPESIFISITY OF T-SPOT.TB IN DETECTING LATENT TUBERCULOSIS IN HEMODIALYSIS PATIENTS. J Respir Indo. 2021;41(1).
9. Lee SSJ, Chou KJ, Dou HY, Huang TS, Ni YY, et al. High Prevalence of Latent Tuberculosis Infection in Dialysis Patients Using the Interferon- Gamma- Release Assay and Tuberculin Skin Test. Clinical Journal of the American Society of Nephrology. 2010; 5: 1451–1457
10. Ahmadinejad Z, Ardalan FA, Safy S, and Nikbakht G. Diagnosis of Latent Tuberculosis Infection in Candidates for Kidney Transplantation (Comparison of Two Tests). Acta Medica Iranica. 2012; 50(5).
11. Sester M, Sester U, Clauer P, Heine G, Mack U, et al. Tuberculin skin testing underestimates a high prevalence of latent tuberculosis infection in hemodialysis patients. Kidney International. 2004; 65:1826-1834.
12. Al Jahdali H, Ahmed AE, Balkhy HH, Baharoon S, Al Hejaili FF, et al. Comparison of the tuberculin skin test and Quanti-FERON-TB Gold In-Tube (QFT-G) test for the diagnosis of latent tuberculosis infection in dialysis patients. Journal of Infection and Public Health. 2013; 6:166-172.
13. Kim SH, Lee SO, Park JB, Park IA, Park SJ, et al. A Prospective Longitudinal Study Evaluating the Usefulness of a T-Cell-Based Assay for Latent Tuberculosis Infection in Kidney Transplant Recipients. American Journal of Transplantation. 2011; 11: 1927-1935.
14. Al Wakeel JS, Makoshi Z, Al Ghonaim M, Al Harbi A , Al Suwaida A, et al. The use of Quantiferon-TB gold in-tube test in screening latent tuberculosis among Saudi Arabia dialysis patients. Annals of Thoracic Medicine. 2015; 10(4).
15. Lee SH, Kim HJ, Park SJ, Kim TH, Park SJ, et al. Serial interferon-gamma release assays for latent tuberculosis in dialysis patients with end stage renal disease in a Korean population. BMC Infectious Diseases.2015;15:381.
16. Ates G, Yildiz T, Danis R, Akyildiz L, Erturk B, et al. Incidence of Tuberculosis Disease and Latent Tuberculosis Infection in Patients with End Stage Renal Disease in an Endemic Region. Renal Failure. 32:1, 91-95.
17. Chung WK, Zheng ZL, Sung JY, Kim S, Lee HH, et al. Validity of interferon-c-release assays for the diagnosis of latent tuberculosis in haemodialysis patients. Clin Microbiol Infect. 2010; 16: 960–965.
18. Shu CC, Tsai MK, Lin SW, Wang JY, Yu CY, et al. Latent Tuberculosis Infection Increases in Kidney Transplantation Recipients Compared With Transplantation Candidates: A Neglected Perspective in Tuberculosis Control. CID. 2019:71.
19. Agarwal SK, Singh UB, Zaidi SH, Gupta S, and Pandey RM. Comparison of interferon gamma release assay & tuberculin skin tests for diagnosis of latent tuberculosis in patients on maintenance haemodialysis. Indian J Med Res. 2015; 141: 463-468.
20. Sultan KM, Obaidy MW and Hasan AM. Prevalence of Latent Tuberculosis in End Stage Renal Disease Patients at Baghdad Teaching Hospital. Tuberculosis renal disease the iraqi postgraduate medical journal. 2016; 15(3).
21. Hassan HAD, Shorman M, Housawi AREI and Elsammak MY. Detecting Latent Tuberculosis Infection Prior to Kidney Transplantation in a Tertiary Hospital in Saudi Arabia: Comparison of the T-SPOT.TB Test and Tuberculin Test. British Microbiology Research Journal . 2013;3(2): 116-127.
22. Chagas AC, Hans Filho G, de Oliveira SM, Ivo ML, Corrêa Filho RA, Donatti MI. Prevalence of latent tuberculosis and treatment adherence among patients with chronic kidney disease in Campo Grande, State of Mato Grosso do Sul. Revista da Sociedade Brasileira de Medicina Tropical. 2014;47(2):204-11.
23. Romanowski K, Rose C, Cook VJ, Sekirov I, Morshed M, Djurdjev O, et al. Effectiveness of Latent TB Screening and Treatment in People Initiating Dialysis in British Columbia, Canada. Canadian journal of kidney health and disease. 2020;7.
24. Hussein MT, Yousef LM and Ali AT. Detection of latent tuberculosis infection in hemodialysis patients: Comparison between the quantiferon-tuberculosis gold test and the tuberculin skin test. Egyptian Journal of Bronchology. 2017; 11(3).
25. Shankar MSR, Aravindan AN, Sohal PM, Kohli HS, Sud K, et al. The prevalence of tuberculin sensitivity and anergy in chronic renal failure in an endemic area: tuberculin test and the risk of post-transplant tuberculosis. Nephrol Dial Transplant. 2005; 20: 2720–2724.
26. Triverio PA, Bridevaux PO, Lombard PR, Niksic L, Rochat T, et al. Interferon-gamma release assays versus tuberculin skin testing for detection of latent tuberculosis in chronic haemodialysis patients. Nephrol Dial Transplant. 2009; 24: 1952–1956.
27. Soysal A, Toprak D, Koc M, Arikan H, Akoglu E and Bakir M. Diagnosing latent tuberculosis infection in haemodialysis patients: T-cell based assay (T-SPOT.TB) or tuberculin skin test?. ephrol Dial Transplant. 2012; 27: 1645–1650.
28. Habesoglu MA, Torun D, Demiroglu YZ, Karataslı M, Sen N, et al. Value of the Tuberculin Skin Test in Screening for Tuberculosis in Dialysis Patients. Transplantation Proceedings. 2007; 39:883–886.
29. Hoffmann M, Tsinalis D, Vernazza P, Fierz W, and Binet I. Assessment of an Interferon-g release assay for the diagnosis of latent tuberculosis infection in haemodialysis patients. Swiss Med Wkly. 2010;140(19–20):286–292.
30. Agarwal SK, Gupta S, Bhowmik D and Mahajan S. Tuberculin skin test for the diagnosis of latent tuberculosis during renal replacement therapy in an endemic area: A single center study. Indian Journal of Nephrology. 2010; 20(3).
31. Lee SSJ, Chou KJ, Su IJ, Chen YS, Fang HC, et al. High Prevalence of Latent Tuberculosis Infection in Patients in End-Stage Renal Disease on Hemodialysis: Comparison of QuantiFERON-TB GOLD, ELISPOT, and Tuberculin Skin Test. Infection. 2009; 37(2).
32. Lin SY, Chiu YW, Yang HR, Chen TC, Hsieh MH, et al. Association of vitamin D levels and risk of latent tuberculosis in the hemodialysis population. Journal of Microbiology, Immunology and Infection. 2020; 06(001).
33. Baek SD , Jeung S and and Kang JY. Nutritional Adequacy and Latent Tuberculosis Infection in End-Stage Renal Disease Patients. Nutrients. 2019; 11: 2299.
34. Bandiara R , Sukesi L , Indrasari A , Kulsum ID and Rudiansyah M. The Relationship between Nutritional Status and Latent Tuberculosis in Routine Hemodialysis. Open Access Maced J Med Sci. 2021; 17; 9(B):945-951.
35. Ogawa Y, Harada M, Hashimoto K and Kamijo Y. Prevalence of latent tuberculosis infection and its risk factors in Japanese hemodialysis patients. Clinical and Experimental Nephrology. 2021.
36. SAYARLIOĞLU H, GÜL M, DAĞLI CE, DOĞAN E, ŞAHİN M, et al. QuantiFERON-TB Gold test for screening latent tuberculosis infection in hemodialysis patients. Tüberküloz ve Toraks Dergisi. 2011; 59(2): 105-110.
37. Ibarra LC, Estrada JJT, García GM, and Santiago JC. Infección latente por Mycobacterium tuberculosis y adherencia al tratamiento en una cohorte de pacientes pretrasplante renal. Revista Mexicana de Trasplantes. 2017; 6(2).
38. Seyhan EC, So¨ku¨cu¨S, Altin S, Gu¨nlu¨og˘lu G, Trablus S, et al. Comparison of the QuantiFERON-TB Gold In-Tube test with the tuberculin skin test for detecting latent tuberculosis infection in hemodialysis patients. Transplant Infectious Disease. 2010;12: 98-105.
39. Wauters A, Peetermans WE, Van den Brande P, De Moor B, Evenepoel P, et al. The value of tuberculin skin testing in haemodialysis patients. Nephrol Dial Transplant. 2004; 19: 433–438.
40. Fang CH, Chou KJ, Chen CL, Lee PT, Chiou YH, et al. Tuberculin Skin Test and Anergy in Dialysis Patients of a Tuberculosis-Endemic Area. Nephron. 2002;91:682–687.
41. Winthrop KL, Nyendak M, Calvet H,Oh P, Lo M, et al. Interferon-Gamma-Release Assays for Diagnosing Mycobacterium tuberculosis Infection in Renal Dialysis Patients. Clin J Am Soc Nephrol. 2008; 3: 1357–1363.
42. Woeltje KF, Mathew A, Rothstein M, Seiler S and Fraser VJ. Tuberculosis Infection and Anergy in Hemodialysis Patients. American Journal of Kidney Diseases. 1998; 31(5): 848-852.
43. Smirnoff M, Patt G, Seckler R and Adler JJ. Tuberculin and Anergy Skin Testing of Patients Receiving Long-term Hemodialysis. CHEST. 1998; 113 (1).
44. Connell DW, Singanayagam A, Charif R, George PM, Molyneaux PL, et al. A comparison between interferon gamma release assays and the tuberculin skin test in the contact tracing of patients wit. h chronic kidney disease. Thorax. 2011;66:729e730.
45. Cengiz K and Sxeker A. Boosted tuberculin skin testing in hemodialysis patients. Am J Infect Control. 2006; 34(6).
46. Akcay A, Erdem Y, Altun B, Usalan C, Agca E, et al. The booster phenomenon in 2-step tuberculin skin testing of patients receiving long-term hemodialysis. Am J Infect Control. 2003;31:371-4.
47. Dogan E, Erkoc R, Sayarlioglu H and Uzun K. Tuberculin Skin Test Results and the Booster Phenomenon in Two-Step Tuberculin Skin Testing in Hemodialysis Patients. Renal Failure. 2005; 27(4): 425-428.
48. Foster R, Ferguson TW, Rigatto C, Lerner B, Tangri N and Komenda P. A retrospective review of the two-step tuberculin skin test in dialysis patients. Canadian Journal of Kidney Health and Disease. 2016; 3:28
49. Khosroshahi HT, Shojaie EA, Habibzadeh D and Hajipour B. Comparison of 5 IU and 10 IU Tuberculin Test Results in Patients on Chronic Dialysis. Saudi J Kidney Dis Transpl. 2012;23(4):823-826
50. Altunoren O, KahramaHn, Sayarlıoğlu H, Yavuz YC, Doğan E and Köksal N. The Affecting Factors and Comparison of Tuberculin Skin Test in Peritoneal Dialysis and Hemodialysis Patients. Renal Failure. 2012;34(3): 304-307.
51. Edathodu J, Varghese B, Alrajhi AA, et al. Diagnostic potential of interferon-gamma release assay to detect latent tuberculosis infection in kidney transplant recipients. Transpll Infect Dis. 2017; 19(2).
52. Maciel MMMD, Ceccato MG, Carvalho WS, et al. Prevalence of latent Mycobacterium tuberculosis infection in renal transplant recipients. J Bras Pneumol. 2018;44(6):461-468.
53. Igari H, Akutsu N, Ishikawa S, Aoyama H, Otsuki K, et al. Positivity rate of interferon-g release assays for estimating the prevalence of latent tuberculosis infection in renal transplant recipients in Japan. J Infect Chemother. 2019; 25: 537e542.
54. Meinerz G, da Silva CK, Dorsdt DMB, Adames JB, Andrade JP, et al. Latent tuberculosis screening before kidney transplantation in the South of Brazil. J. Bras. Nefrol. 2021;43(4):520-529.
55. GrantJ, Jastrzebski J, Johnston J, et al. Interferon-gamma release assays are a better tuberculosis screening test for hemodialysis patients: A study and review of the literature. Can J Infect dis Med Microbiol. 2012;23(3):114-116.35.
56. Chung WK, Zheng ZL, Kim HS, Park JW, Lee HJ, et al. Serial testing of interferon-g-release assays for the diagnosis of latent tuberculosis in hemodialysis patients. Journal of Infection. 2010; 61, 144e149
57. Mohtashami AZ, Amiri A, Hadian B and Nasiri P. Assessment of the prevalence of latent tuberculosis infection in hemodialysis patients using tuberculin skin test. J Renal Inj Prev. 2022; 1.
58. Wang PH, Lin SY, Lee SSJ, Lin SW, Lee CY, et al. CD4 response of QuantiFERON‑TB Gold Plus for positive consistency of latent tuberculosis infection in patients on dialysis. Scientific Reports. 2020; 10:21367.
59. Harris M, Johnston J and Ronald L. Latent Tuberculosis Treatment Cascade in Chronic Kidney Disease Patients: The Vancouver Experience. OFID. 2016:3 (Suppl 1).
